# Hierarchical repeating earthquakes with strong downward directivity

**DOI:** 10.1126/sciadv.aeb3563

**Published:** 2026-09-18

**Authors:** Dawei Gao, Satoshi Ide

**Affiliations:** ^1^ Key Laboratory of Ocean Observation and Forecasting, Laboratory of Marine Geology and Environment, Institute of Oceanology, Chinese Academy of Sciences, Qingdao 266000, China.; ^2^ Laboratory for Marine Geology, Qingdao National Laboratory for Marine Science and Technology, Qingdao 266000, China.; ^3^ Department of Earth and Planetary Science, The University of Tokyo, Tokyo 113-8654, Japan.

## Abstract

How and to what extent earthquakes repeat remain key open questions with important implications for early warning and hazard forecasting. Using a 19-year waveform dataset and a starting catalog of repeating earthquakes (repeaters) from small segments (<1%) of the Tohoku-Hokkaido megathrust, we quantify rupture repeatability for ∼M3-5.5 repeaters. We observe strong spatial clustering of hypocenters and centroids, widespread and persistent hierarchical fault structures, systematic downdip hypocenter-centroid directivity that may amplify coastal shaking, and unexpectedly limited rupture repeatability among events classified as repeaters. To interpret these patterns, we propose a hierarchical fault model and define “hierarchical repeaters” that link two end-members with similar initiation (hypocenter repeaters) and similar termination (centroid repeaters). Our results highlight geometric controls on rupture recurrence and suggest intrinsic limits on predictability within the spatially restricted, preselected sample analyzed here. If earthquakes are fundamentally self-similar, hierarchical geometric controls may operate across scales, from small repeaters to giant megathrust earthquakes.

## INTRODUCTION

Megathrust faults in subduction zones are capable of producing the largest earthquakes on Earth, often generating tsunamis and posing serious threats to coastal populations (*1*–*3*). However, knowledge of such megathrust events remains limited. Their multicentury recurrence intervals (*4*–*6*), together with the short duration of modern seismic records (*7*), hinder direct observation of multiple earthquake cycles and challenge efforts to assess rupture predictability. In contrast, smaller repeating earthquakes (repeaters) (M <6) on the plate interface recur every few months to years (*8*), offering a unique natural laboratory to investigate the physical conditions governing repeated rupture. Characterizing their source structure and recurrence behavior may yield key insights into the mechanisms that control earthquake nucleation and long-term rupture predictability across scales.

The fault segment hosting repeaters is generally considered structurally simple in classical asperity models, comprising a fully locked patch with uniform rupture properties embedded within a creeping background (*8*). However, several case studies have suggested that the source regions of such events may be more structurally complex, exhibiting a persistent hierarchical fault structure (*9*–*12*). One notable example is the Naka-Oki sequence off the coast of Ibaraki, eastern Japan, which includes colocated large (∼M5) and small (∼M3) repeaters (*10*). Despite the substantial difference in magnitude, these colocated events are characterized by near-identical initial waveforms, implying a hierarchical rupture growth process (*10*). Building on this observation, two recent studies systematically compared the initial seismic waveforms of events with varied magnitudes in the Japan Trench and northern California, suggesting that hierarchical fault structures are a common feature of mature faults (*13*, *14*). Nonetheless, the prevalence of such structures in repeater source regions and their influence on rupture repeatability remain poorly understood.

Research on the recurrence behavior of repeaters also remains limited primarily because of their generally low magnitudes and the lack of high-quality observations. Insights have been derived mostly from well-instrumented regions, such as Parkfield and the Tohoku subduction zone, and are based largely on case studies. Limited applications of slip inversions and rupture directivity analyses suggest consistent rupture dimensions (*11*, *15*–*17*) and propagation directions (*18*, *19*). However, comparing rupture characteristics across events remains challenging when relying on event-specific approaches, such as direct slip inversion, because of variability in data coverage and quality (*20*, *21*). A new network correlation coefficient (NCC) method proposed by Chang and Ide (*22*) simplifies the rupture analysis and enables high-precision relative location of hypocenters and centroids of multiple events within a unified coordinate system, thereby providing first-order constraints on rupture size and direction. On the basis of three case studies, they show that while repeaters can exhibit tightly clustered hypocenters or centroids, their rupture behaviors may differ substantially. These observations give rise to the concepts of hypocenter repeaters and centroid repeaters, in which events resemble each other at the onset or termination of rupture, respectively. Although the latter are commonly regarded as repeating earthquakes, both represent end-members of complex yet similar rupture processes. Despite these advances, a systematic and quantitative evaluation of earthquake rupture repeatability remains absent.

**Fig. 1.**
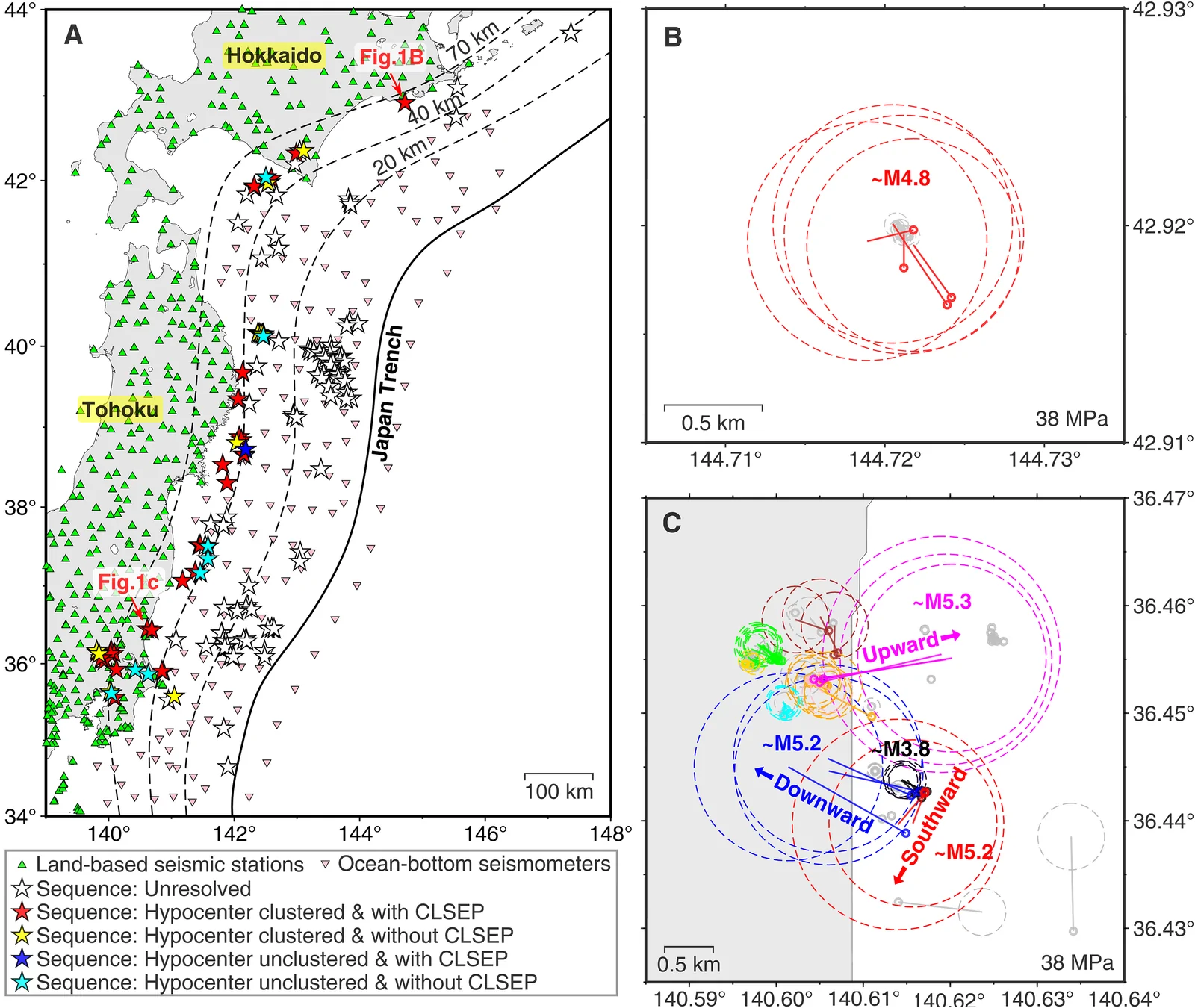
Distribution of target potential repeating sequences and located examples. (**A**) Spatial distribution of the 150 target potential repeating sequences. (**B** and **C**) Examples of located sequences. Different colors indicate distinct families of identified repeaters, except for gray, which denotes anchor events (M <3.2) or other background earthquakes (i.e., nonrepeaters). Small solid circles mark hypocenter locations, and large dashed circles indicate nominal rupture areas, calculated with an SD of 38 MPa (*9*, *22*, *25*–*27*). Solid lines connect each hypocenter to its corresponding centroid. Note that some hypocenters fall outside their associated rupture areas, likely reflecting the fact that the actual rupture geometry may be elliptical and elongated along the rupture direction, whereas a circular rupture model is assumed for simplicity (*22*, *27*).

To address the aforementioned questions, we present a large-sample NCC-based statistical study of hypocenter-centroid behavior and rupture repeatability for M ≥ 3.2 repeaters along the Tohoku-Hokkaido megathrust based on 19 years (1 April 2004 to 31 December 2022) of high-quality waveform data (Fig. 1A). The combination of abundant land-based stations, including the dense Hi-net borehole array, and ocean-bottom seismometers (S-net), together with a cutting-edge hypocenter-centroid relative relocation (*22*), enables detailed statistical analyses of source structure and rupture features of interplate repeaters. Importantly, we introduce a uniform, quantitative framework for measuring rupture repeatability (Materials and Methods) that is applicable across the full dataset. This framework enables consistent comparisons across rupture attributes, including hypocenter and centroid locations, rupture dimensions, and propagation directions, thereby moving beyond prior case-based qualitative studies. Our results highlight the geometric control on rupture repeatability and may shed new light on the recurrence behavior of megathrust ruptures.

## RESULTS

### Target sequence selection and relocation

We begin the experiment by selecting 150 interplate ∼M4.5 potential repeating sequences from a recent study (Materials and Methods) (*23*). As these events were identified on the basis of waveform similarity and S-P differential time without relocation (*23*), their absolute location uncertainties can reach several tens of kilometers (fig. S1A). To improve the initial locations, we applied HypoDD (*24*) to each sequence, reducing relative location uncertainties to generally less than 100 m (Materials and Methods) (fig. S1B).

To investigate the source structure and rupture characteristics of repeaters, we apply the state-of-the-art NCC hypocenter-centroid relative relocation method (*22*), which uses small-magnitude earthquakes as anchors by merging their hypocenters and centroids into single hypocentroids to constrain the relative positions of hypocenters and centroids of larger events (fig. S2) (Materials and Methods). For each target sequence, we search for nearby earthquakes within 2 km horizontally and 3 km vertically, with magnitudes ≥2.0 (*22*), yielding a total of 4127 events. Sequences separated by less than these thresholds are merged to avoid duplication. Following prior work, we use events with M < 3.2 as anchors (*22*) to perform hypocenter-centroid relocation for each individual sequence, as their source dimensions are comparable to the sub–100 m uncertainties in location estimates (Materials and Methods). Ultimately, we resolve 45 sequences, each containing at least one event with M ≥ 4.5, comprising a total of 2435 earthquakes. This includes 747 events with M ≥ 3.2 and 175 with M ≥ 4.5, located beneath onshore and nearshore regions, primarily downdip of the seismogenic zone at depths greater than 40 km (Fig. 1A) (Materials and Methods). Notably, the 175 resolved M ≥ 4.5 earthquakes represent ∼10% of similar-sized and above events along the Tohoku-Hokkaido megathrust during the same period, although our analysis is confined to less than 1% of the subduction interface.

### Prevalent repeating earthquakes

Here, we apply a robust repeater identification procedure to the 747 events with M ≥ 3.2, whose rupture dimensions exceed the sub–100 m location uncertainty (fig. S3). Assuming a stress drop (SD) of 38 MPa appropriate for the study area (*9*, *22*, *25*–*27*), we identify 156 repeating families comprising 484 earthquakes (M3.2 to M5.4) (Materials and Methods and data S1). Although repeaters typically constitute only a small fraction of seismicity in active fault zones (*23*, *28*–*30*), we obtain a high proportion here (65%, 484 of 747) because we start from an a priori repeater catalog: More than 90% of our repeaters fall within the (*23*) catalog for the same spatiotemporal window and magnitude range. Assuming widely used but much smaller SDs (e.g., 10, 3 MPa) (*31*) increases this proportion up to 75% (Materials and Methods). The results indirectly suggest predominantly stable creep within the previously identified repeater-rich regions (*23*).

### Source structure and rupture characteristics

We find that repeaters within the same family can initiate from distinct locations (Fig. 1, B and C), indicating hidden complexity in their rupture processes. Conversely, repeaters with substantial magnitude differences (MDs) may originate from nearly identical spots, suggesting hierarchical rupture growth (e.g., the ∼M3.8 and ∼M5.2 families shown in black and red, respectively, in Fig. 1C). Similar to the previously reported Naka-Oki M3 and M5 repeaters (*10*, *11*), these colocated events exhibit nearly identical initial waveforms (fig. S4). Furthermore, variations in rupture propagation are observed both within the same repeater family and among different families in localized regions (Fig. 1, B and C). These patterns are unlikely to be explained solely by the bimaterial effect (*32*, *33*) at such small spatial scales. A more plausible explanation is the control of small-scale fault-zone heterogeneity possibly associated with geometric complexity.

To systematically quantify the source structure and rupture characteristics of the 45 well-resolved sequences, we focus our analysis on four key aspects.

#### Rupture initiation and termination

To test whether rupture onset and termination, represented by hypocenters and centroids, respectively, are spatially random, we apply the nearest-neighbor test (NNT) to each sequence (Materials and Methods). The NNT quantifies departures from a Poisson point process by comparing the observed mean nearest-neighbor distance ${\bar{r}}_{\text{obs}}$ with its theoretical expectation ${\bar{r}}_{\text{exp}}$ (*27*, *34*). Following (*27*), we approximate the study area using the minimum enclosing circle of all hypocenters or centroids within each sequence. With this definition, a total of 80% (36 of 45) and 76% (34 of 45) of the sequences exhibit statistically significant clustering of hypocenters and centroids (Fig. 1 and fig. S5), respectively, at the 95% confidence level. These results suggest that rupture nucleation and arrest are spatially nonrandom and likely shaped by persistent fault heterogeneities linked to geometrical irregularities (*35*, *36*).

However, our sequence sizes span a wide range (<20 to >300 events), and 73% (33 of 45) contain ≥20 events (fig. S6), necessitating a careful assessment of sample-size effects on the NNT statistics: When sequence size η is small, particularly for η < 20, statistically significant departures from randomness require larger deviations of the clustering ratio $R={\bar{r}}_{\text{obs}}/{\bar{r}}_{\text{exp}}$ from unity (with *R* < 1 indicating clustering, whereas *R* > 1 suggests a tendency toward regular spacing) (*27*, *37*). As expected from these sample-size effects, fig. S7A shows that in the small-sample regime (η < 20), *R* values scatter around unity for both hypocenters and centroids, including a few cases with *R* > 1, while *Z* scores (evaluating statistical significance) generally remain low and often fall below the 95% significance threshold (*Z* = 1.96). As η increases beyond ∼20, *R* becomes systematically <1 (with only a weak further decrease with η), whereas *Z* increases strongly with η for both hypocenters and centroids, reflecting improved statistical power and increasingly robust evidence for clustering. To further evaluate the influence of small-η sequences, we progressively exclude sequences below a minimum event threshold (increment = 5) and recompute the fraction of clustered sequences; the clustered fraction increases monotonically for both hypocenters and centroids and exceeds 90% once η ≥ 20 (fig. S8A), demonstrating that our clustering inference is robust. Because some sequences can be highly elongated along strike, a circular area approximation may introduce geometry-related boundary effects; we therefore repeat the analysis using a minimum enclosing ellipse. The results remain broadly comparable (figs. S7B and S8B), and within the well-resolved regime (η ≥ 20), the fraction of clustered sequences remains >80%, ∼10% lower than that obtained using the circular area assumption (fig. S8).

#### Hierarchical rupture growth

**Fig. 2.**
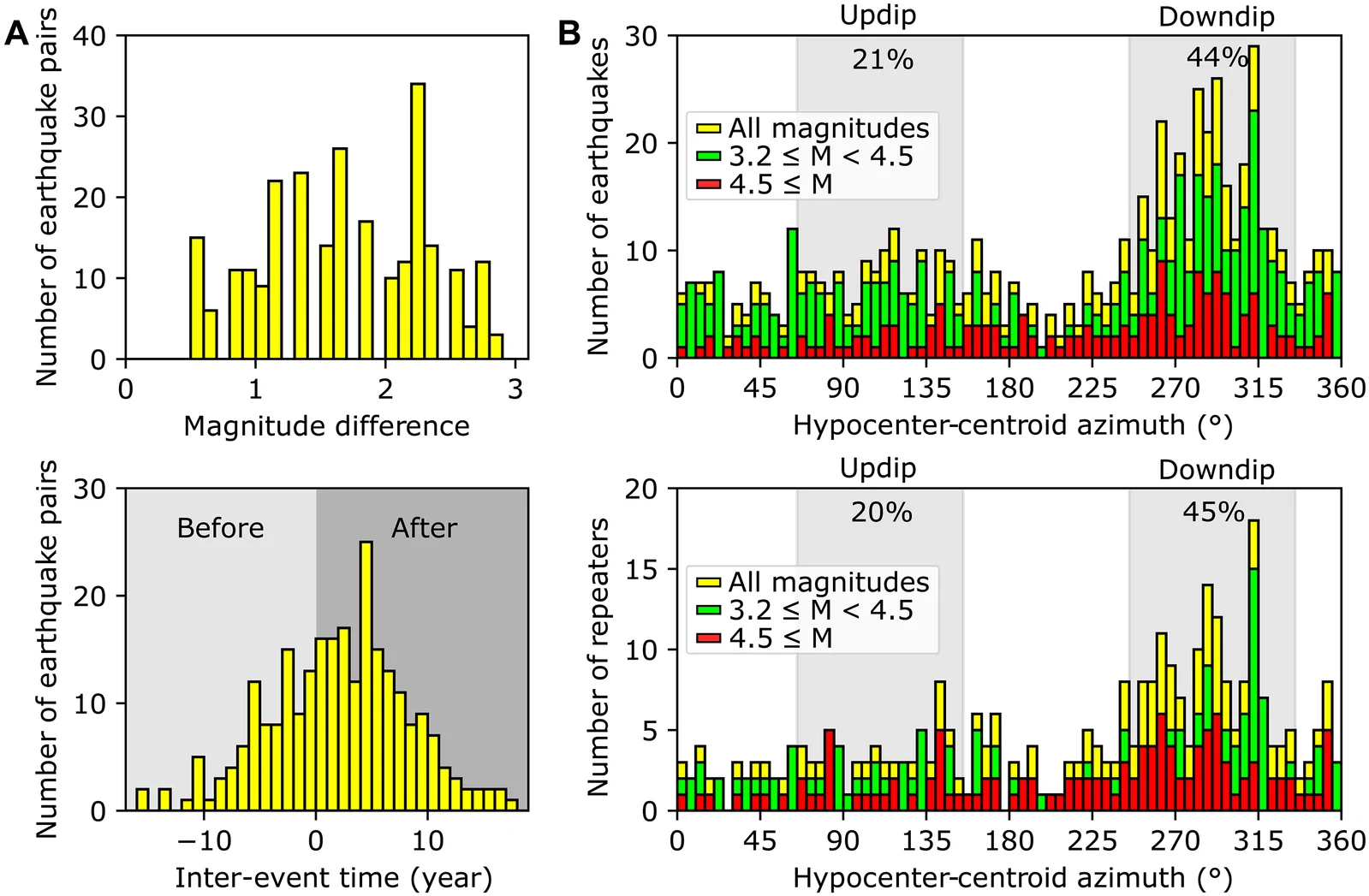
Statistical properties of CLSEPs and rupture azimuths. (**A**) Histograms of MDs (top) and inter-event times (bottom) for CLSEPs. MDs are computed as absolute values and are therefore independent of event ordering within each pair. In the bottom panel, the inter-event time is defined as *t*_small_ − *t*_large_: Negative (positive) values indicate that the small earthquake occurred before (after) the corresponding large event. (**B**) Histograms of HC azimuths for all earthquakes (top) and identified repeaters (bottom). Repeater families are identified with an SD of 38 MPa (*9*, *22*, *25*–*27*). Events (top) or repeater families (bottom) with HC distances less than 100 m are excluded from the azimuth analysis. In both panels, gray shading denotes the updip and downdip rupture azimuth ranges.

Given the limited magnitude range of our dataset (M2 to M6), we follow prior work (*13*) and classify events with M ≥ 4.5 and M ≤ 4.0 as large and small earthquakes, respectively. Since the analyzed events are interplate earthquakes, we identify colocated large-small earthquake pairs (CLSEPs) based on a horizontal interhypocenter distance of less than 100 m (*10*, *13*, *14*), allowing each large earthquake to be associated with multiple smaller ones. CLSEPs are observed in 60% (27 of 45) of the sequences and involve 40% (70 of 175) of the M ≥ 4.5 events, resulting in a total of 254 pairs with an average MD of 1.68 (data S2). These include 43 pairs of 0.5 ≤ MD < 1.0, 54 pairs of 1.0 ≤ MD < 1.5, 58 pairs of 1.5 ≤ MD < 2.0, 73 pairs of 2.0 ≤ MD < 2.5, and 26 pairs of 2.5 ≤ MD < 3.0 (Fig. 2A, top). We note that the 40% occurrence rate of large earthquakes associated with at least one smaller event is substantially higher than the 20% previously reported (*13*) based on waveform similarity analysis. This increased proportion may reflect more hierarchical structures in the source regions of repeaters or, alternatively, the use of more inclusive criteria compared to waveform-based methods.

The inter-event times of CLSEPs are typically on the order of several years, with a pronounced tendency for small events to follow large ones (Fig. 2A, bottom). When only the nearest small event occurring before and after each large earthquake is considered, the distribution becomes approximately symmetric, with few events occurring immediately adjacent to the large one (fig. S9, B to D). The broad temporal distribution (Fig. 2A and fig. S9B) suggests that the paired events are not directly causally related but are instead driven by long-term stress accumulation associated with plate subduction, a process consistent with the mechanism of repeaters. These observations imply the presence of persistent fault structures capable of repeatedly hosting both large and small events (i.e., hypocenter repeaters). The observed temporal asymmetry in Fig. 2A may reflect a triggering effect, wherein small earthquakes are more readily activated by stress perturbations from neighboring ruptures than their larger counterparts.

#### Rupture directivity

Although some rupture details may be lost, the hypocenter-to-centroid (HC) direction offers a first-order constraint on rupture directivity (*22*). Given our sub–100 m location uncertainty, we restrict the rupture directivity analysis to events with rupture dimensions larger than this (i.e., M ≥ 3.2) and exclude those with HC distances <100 m, yielding 639 qualified events. Considering that the relative plate motion between the Pacific and North American plates in the Tohoku-Hokkaido region is oriented ∼70° northwest (*38*), we define updip (i.e., seaward) and downdip (i.e., landward) rupture azimuths as 110° ± 45° and 290° ± 45°, respectively. The HC azimuth distribution is strongly nonuniform, indicating a preferred rupture direction (Rayleigh test *P* = 3.95 × 10^−15^; circular mean = 295.87°; circular variance = 0.77): 44.3% of events fall within the downdip sector, compared with 21.1% in the updip sector [bootstrap 95% confidence intervals (CIs): 40.4 to 48.0% and 18.0 to 24.3%, respectively] (Fig. 2B and fig. S10A). Moreover, the color-coded, magnitude-binned histograms suggest a broadly similar azimuthal pattern across magnitude ranges (3.2 ≤ M < 4.5 and M ≥ 4.5; Fig. 2B). The same downdip preference is also observed for repeaters, which show significant nonuniformity (Rayleigh test *P* = 3.64 × 10^−10^; circular mean = 284.52°; circular variance = 0.74), with 45.3% downdip versus 20.2% updip (bootstrap 95% CIs: 40.1 to 50.6% and 15.8 to 24.5%) (Fig. 2B and fig. S10B), suggesting that the downdip tendency is shared by both the overall catalog and the repeater subset. To explicitly account for azimuth errors induced by location uncertainty, we propagate horizontal location errors for both hypocenters and centroids through Monte Carlo (MC) perturbations (10,000 realizations). In our baseline model, independent Gaussian errors are added to the east and north components (σ_*xy*_ = 40 m per component, corresponding to a 95% horizontal/radial uncertainty of ∼100 m) (Materials and Methods). The nonuniformity and downdip preference remain significant in 100% of realizations for both all events and repeaters (fraction of realizations with *P* < 0.05 = 1.000; Rayleigh *P* value 97.5th percentiles: 1.75 × 10^−13^ and 7.60 × 10^−9^, respectively), demonstrating robustness to plausible measurement uncertainty (Materials and Methods). Results remain essentially unchanged under more conservative, upper-bound perturbations (Materials and Methods), confirming that the inferred predominant rupture direction is not an artifact of the assumed location-error amplitude.

In contrast, (*39*) reported prevalent updip directivity in approximately the same region based on azimuthal variations of apparent rupture durations estimated from empirical Green’s function (EGF)–derived apparent moment rate functions (AMRFs). While a comprehensive evaluation of their full dataset is beyond the scope of this study, we reexamined a limited number of representative examples (one in detail and four qualitatively) and find that, in those cases, directivity estimates can be sensitive to station subset and data quality, potentially contributing to discrepant azimuthal trends (Materials and Methods and fig. S11). As an additional conservative sensitivity check, we further exclude events with HC distances up to 500 m (five times our nominal sub–100 m uncertainty) and replot the color-coded, magnitude-binned azimuth histograms. The downdip-dominant pattern remains qualitatively unchanged (fig. S12). A similar downdip preference for similar-sized events has also been reported in northern Chile subduction zone (*40*), suggesting a broader relevance of this rupture style. Notably, downward rupture propagation immediately after nucleation may amplify ground motion and elevate seismic hazard in coastal regions (*2*). Such behavior has also been documented in many large earthquakes in the study area, including the 1952 moment magnitude (*M*_w_) 8.2, 1968 *M*_w_ 8.2, and 2003 *M*_w_ 8.1 Tokachi-oki (*41*, *42*); 1978 *M*_w_ 7.4 and 2005 *M*_w_ 7.2 Miyagi-Oki (*43*); 1989 *M*_w_ 7.4 Iwate-Oki (*44*); 1994 *M*_w_ 7.7 Sanriku-Oki (*45*); 2008 M7 Ibaraki-Oki (*46*); and 2011 *M*_w_ 9.0 Tohoku-Oki (*2*) events, although some ruptures may exhibit upward propagation in later stages, as observed in the 2011 Tohoku-Oki earthquake (*2*).

##### Rupture repeatability

The rupture repeatability of repeaters remains poorly understood, with no quantitative assessment currently available. To address this gap, we introduce a rupture similarity index (RSI), which is constructed by transforming the within-family scatter of each metric into a comparable similarity score bounded in [0,1], with higher values indicating greater consistency in rupture characteristics (Materials and Methods). The underlying normalizations of RSI are chosen to reflect the natural scale of each measurement (circular variability for azimuths, relative spread for HC distances, and a rupture-length scale for locations), enabling a consistent interpretation across rupture attributes (Materials and Methods). In this framework, a repeater family is considered to exhibit repeatable rupture feature if its corresponding RSI exceeds a specified threshold *T* and the threshold acts as a tunable strictness parameter: Higher *T* imposes tighter tolerances and therefore yields fewer families classified as repeatable. Example families spanning low, moderate, and high RSI are presented in fig. S13.

**Fig. 3.**
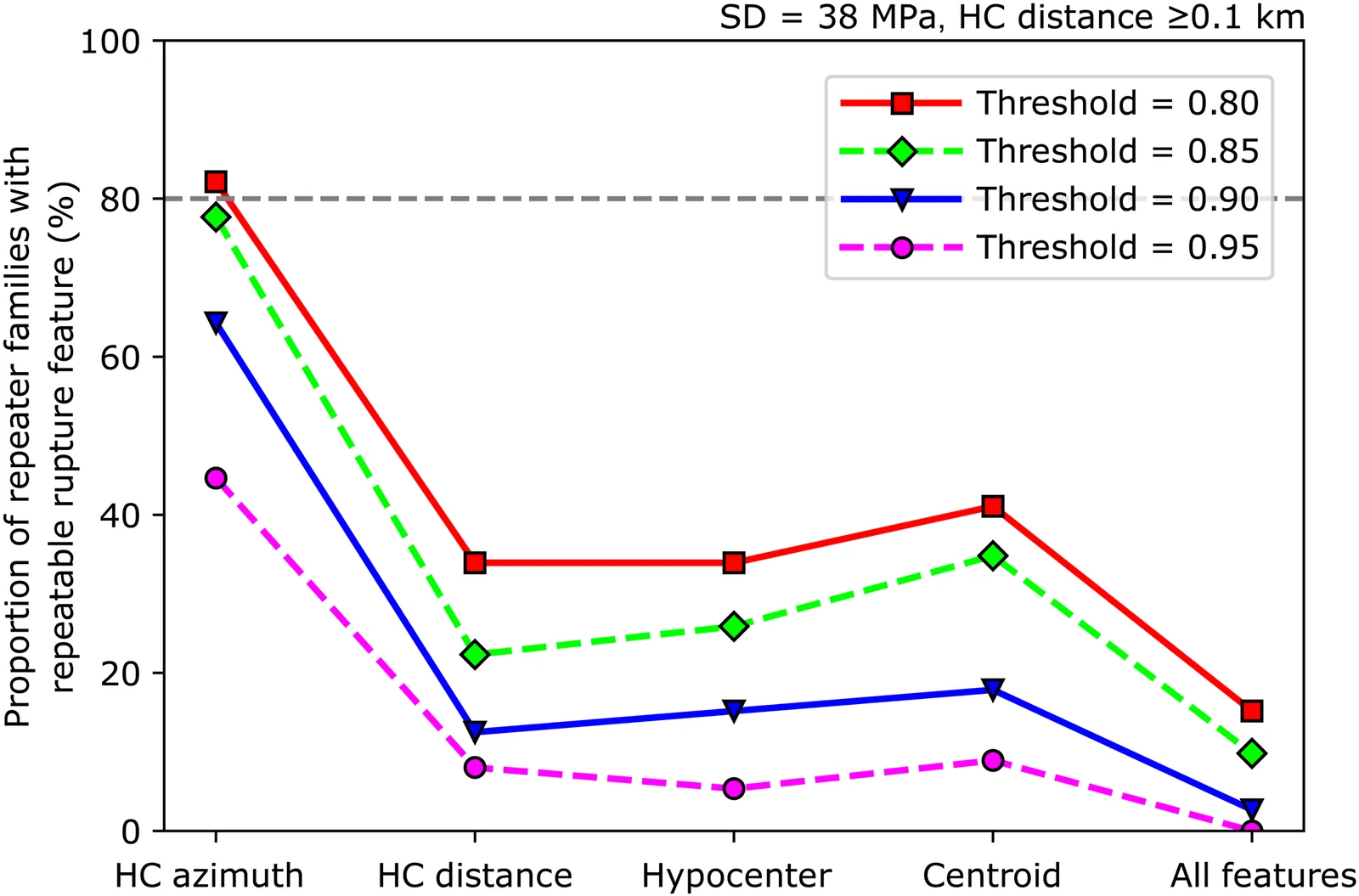
Rupture repeatability of repeaters. Repeater families are identified with an SD of 38 MPa (*9*, *22*, *25*–*27*). Families containing events with HC distances less than 100 m are excluded from the rupture repeatability analysis.

Here, we systematically evaluate rupture repeatability from five aspects: HC azimuth (${\mathrm{RSI}}_{\text{azi}}$), HC distance as a proxy for rupture dimension (${\mathrm{RSI}}_{\text{dist}}$), hypocenter location (${\mathrm{RSI}}_{\text{hypo}}$), centroid location (${\mathrm{RSI}}_{\text{cent}}$), and a composite metric integrating all four features. We define the composite index as the bottleneck similarity across features

$${\mathrm{RSI}}_{\text{comp}}=\text{min}\left({\mathrm{RSI}}_{\text{azi}},{\mathrm{RSI}}_{\text{dist}},{\mathrm{RSI}}_{\text{hypo}},{\mathrm{RSI}}_{\text{cent}}\right)$$

so that a family meets the composite criterion at threshold *T* only when all four feature-specific RSI values are ≥*T*. Considering the location uncertainty, we exclude repeater families that contain events with HC distances <100 m from the rupture repeatability analysis. Assuming a conservative RSI threshold of *T* = 0.80 (Materials and Methods), we find that more than 80% of repeater families show repeatability in HC azimuth, which represents the highest proportion among five aspects. In contrast, only a very limited proportion satisfies the composite criterion and thus exhibit rupture consistency across all four features (Fig. 3). These results suggest that while certain aspects of rupture, such as directivity, tend to repeat, fully repeatable rupture behavior across multiple attributes is relatively rare. Moreover, the proportion of families classified as repeatable decreases with increasing threshold, and under extremely strict criteria (e.g., *T* = 0.95), nearly no families exhibit fully repeatable rupture behavior (Fig. 3). These patterns are robust across a broad range of analysis settings, including the assumed SD (e.g., 3, 10, and 38 MPa) and the HC-distance screening threshold (e.g., 0 to 500 m) (Fig. 3, fig. S14, and table S1). Because range-type dispersion metrics may show some dependence on the number of events, we further assess potential sensitivity to family size. Our repeater families are predominantly small (≤5 events) under all SD assumptions (∼85 to 90% of families). We therefore repeat the analysis after capping the maximum family size at 2, 3, 4, and 5 events, respectively. Across all HC-distance screening criteria and SD assumptions, the results remain generally comparable (fig. S15), indicating a limited effect of family size.

To quantify what level of rupture repeatability is distinguishable given our location uncertainty, we propagate horizontal location errors through ${\mathrm{RSI}}_{\text{comp}}$ using MC perturbations of hypocenter and centroid locations. Similar to the rupture directivity analysis, we add independent Gaussian errors to the east and north components with σ_*xy*_ = 40 m per component, corresponding to an ∼100-m 95% radial uncertainty under an isotropic two-dimensional (2D) error model (Materials and Methods). Following our HC-distance screening (families containing any event with HC distance <100 m are excluded), we compute for each family an exceedance probability (EP) metric, *EP*(*T*), defined as the probability that ${\mathrm{RSI}}_{\text{comp}}$ exceeds a given threshold *T* (fig. S16 and Materials and Methods). The *EP*(*T*) therefore quantifies the joint probability that all four rupture features meet the repeatability criterion under the prescribed location uncertainty. This uncertainty-aware analysis further demonstrates that high confidence, fully repeatable families are exceedingly rare across the tested thresholds (*T* = 0.80 to 0.95). Instead, most families are consistently classified as high-confidence not repeatable, while a minority fall into the indistinguishable category once location uncertainties are propagated. Notably, increasing *T* produces a systematic redistribution from “indistinguishable” to “high-confidence not repeatable” for a range of HC-distance screening thresholds examined, with the indistinguishable fraction approaching zero at the highest *T* values (fig. S16). These results demonstrate that fully repeatable rupture behavior is very limited. Last, we emphasize that our conclusions remain robust across the sensitivity tests performed, including alternative SD assumptions (3 and 10 MPa), HC-distance screening thresholds (0 to 500 m), and substantially larger horizontal perturbation levels (σ_*xy*_ = 80 and 100 m per component), and may provide new insights into the recurrence behavior of megathrust ruptures.

### A hierarchical rupture framework

Given these intriguing observations, what kind of rupture model can account for such complex behaviors? While the classical conceptual asperity model, characterized by spatially distributed slip patches, is sufficient to explain the occurrence of perfect repeaters (*8*, *47*), it falls short in explaining the rupture characteristics revealed in this study, such as the widespread occurrence of CLSEPs and the variable rupture behaviors observed among repeaters within the same family.

**Fig. 4.**
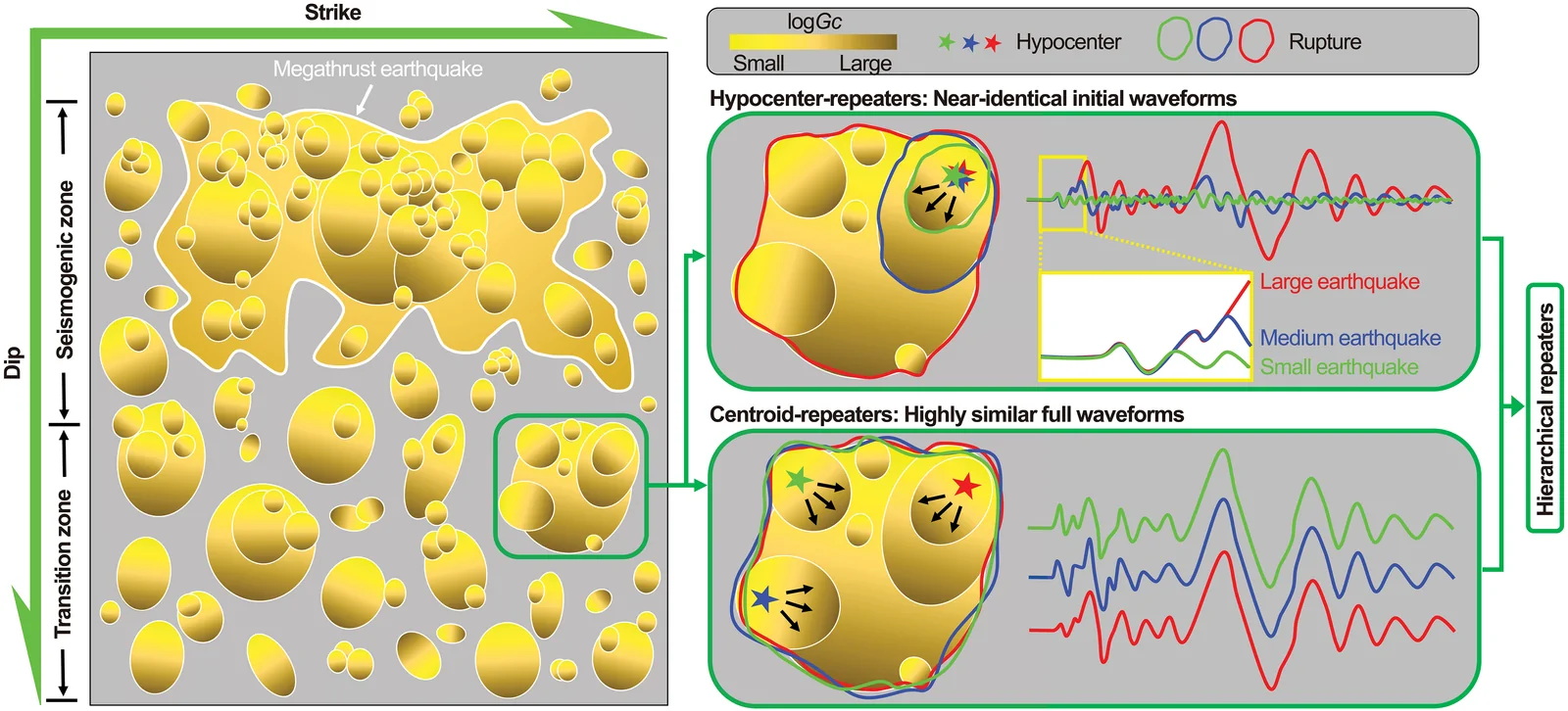
Schematic illustration of a hierarchical patch model producing diverse types of repeaters. The hierarchical organization, represented by variable patch sizes, governs earthquake rupture behavior and controls the extent to which ruptures repeat partially or fully. Large earthquakes may emerge from a cascade-up process initiated by failure on a small patch, with rupture likely propagating downdip.

To overcome these limitations, we advocate for a more comprehensive rupture framework that explicitly incorporates the multiscale geometrical complexity of fault systems, where local variations in geometry render some fault segments more susceptible to slip than others (Fig. 4). This complexity spans multiple scales and is often approximated as fractal (*13*, *14*, *48*–*50*), allowing the fault interface to be idealized as an ensemble of elliptical patches, elongated in the slip direction and distributed fractally (*10*, *11*, *51*–*53*). In this view, multiscale geometry can be mapped onto a corresponding hierarchy in fracture energy (Gc): In the patch models of Ide and Aochi (*51*) and Aochi and Ide (*52*), Gc is explicitly prescribed to increase linearly with patch size. More recently, data-constrained scaling and 3D dynamic simulations further support a near-linear minimum Gc-fault–size relation and show that it enables cascading ruptures across scales (*54*). In our conceptual sketch (Fig. 4), we depict patches as ellipses elongated in the slip direction to reflect rupture directivity; any within-patch, depth-dependent variability is treated as a working hypothesis tied to local geometrical variations rather than an established scaling. In this conceptual picture, rupture may preferentially nucleate on smaller patches and, where geometry permits, within their shallower portions. Larger, stronger patches may embed multiple smaller, weaker ones, forming a hierarchical structure (*9*–*14*, *26*). Under favorable stress conditions, rupture can cascade from small-scale patches into progressively larger ones, with the final earthquake size determined when propagation is arrested at a particular scale by the patch configuration and the surrounding stress field. For fault-slip modelers, this framework can be implemented as a scale-distributed patch ensemble with a near-linear Gc backbone with a depth-dependent term, and tested directly against the observed statistics of rupture initiation versus arrest (hypocenter/centroid clustering), hypocenter-centroid offsets (distance and azimuth), and cascade-like size variability. Notably, the cascading rupture behavior has been explored in numerical simulations (*53*, *54*), providing further support for our hierarchical rupture model.

Within this framework, repeaters can be broadly categorized into two end-member types based on whether rupture similarity is constrained primarily at initiation or termination. Hypocenter-repeaters initiate from nearly identical locations and can be identified either by dense array–based similarity of P-wave onsets (*13*, *14*) or by high-precision relative relocations (*22*); their final magnitudes may vary substantially, reflecting differences in rupture extent. Centroid-repeaters, in contrast, terminate at similar locations and can be robustly identified using physics-based criteria that require substantial overlap of source areas together with small MDs (*12*, *55*–*57*); they typically exhibit comparable magnitudes and high waveform similarity over their full durations, although their initial waveforms may differ slightly because of variations in nucleation locations. We propose a unifying concept of hierarchical repeaters, which encompasses both end-members and reflects the multiscale organization of fault patches that governs rupture nucleation, propagation, and arrest (Fig. 4). In this view, hypocenter- and centroid-repeaters represent different observational expressions of a common hierarchical rupture framework, emphasizing similarity at different stages of rupture evolution.

## DISCUSSION

### Strong downward directivity

In this study, we reveal pronounced downward rupture directivity, which could enhance ground shaking and elevate seismic hazard in coastal regions (*2*). We acknowledge that our NCC relocation method may not fully capture the complexity of rupture behavior in events with intricate source characteristics as it simplifies the rupture process by focusing solely on the hypocenter and centroid locations. However, given that most earthquakes with resolvable directivity are predominantly unilateral [(*58*) and references therein], the NCC method remains sufficient to provide first-order constraints on rupture directivity. Its effectiveness has been extensively validated through multiple approaches (*22*, *27*), including comparisons with slip inversion results for events in the Naka region exhibiting upward and downward rupture directivities (*27*). We also recognize that for events with extremely source complexity, although relatively rare, different methods may capture different aspects of the rupture process and hence yield divergent interpretations. For instance, the 2011 *M*_w_ 9.0 Tohoku earthquake nucleated in the shallow portion of the megathrust, propagated downdip, and subsequently ruptured back updip (*2*). Although both the hypocenter and peak slip were located at shallow depths, strong high-frequency energy was radiated from the downdip region [(*59*) and references therein]. In such cases, methodological differences between our approach and others [e.g., (*39*)] may naturally lead to different interpretations of rupture characteristics.

### Summary and implications

In summary, our study reveals four key findings: (i) Clustered rupture initiation and termination: ∼80% of sequences show statistically significant clustering of hypocenters and centroids at the 95% confidence level, highlighting the deterministic role of local fault heterogeneity in controlling where ruptures start and stop. (ii) Similarity in hierarchical rupture growth: 60% of sequences exhibit CLSEPs within ∼100-m proximity, forming 254 hypocenter-repeater pairs, implying widespread persistent hierarchical fault structures along the plate interface and limited ability to predict earthquake size, even probabilistically. (iii) Strong downward directivity: ∼45% of events with resolvable hypocenter-centroid offsets exhibit pronounced downward rupture propagation, potentially amplifying ground shaking and increasing seismic hazard for coastal cities. (iv) High repeater prevalence in preselected regions but limited rupture repeatability: Across the examined SD values of 3 to 38 MPa, 65 to 75% of M ≥ 3.2 earthquakes from repeater-rich regions fall into repeater families, yet full repeatability across all four RSI features is rare (table S1), revealing hidden complexity among so-called repeaters and challenging the notion of their deterministic behavior.

Together, our study unifies the concepts of hypocenter and centroid repeaters into a generalized framework of hierarchical repeaters (Fig. 4), underscores the critical role of small-scale hierarchical structures in governing rupture dynamics, challenges the classical asperity model (*47*), and establishes a hierarchical rupture framework with important implications for earthquake early warning and probabilistic forecasting. Although so-called repeaters have long been regarded as representative examples of predictable earthquakes (*8*, *13*), our findings demonstrate that their predictability is quite limited because of their diverse rupture behaviors. If earthquakes are fundamentally self-similar (*60*, *61*), the hierarchical geometric controls revealed in our study may operate across scales. Such scale-invariant behavior may link small repeating events with giant megathrust earthquakes.

This hypothesis is supported by observations from the few instrumentally recorded cases of repeated megathrust earthquakes, including the 1952 *M*_w_ 8.2 and 2003 *M*_w_ 8.1 Tokachi-Oki, the 1942 Ms 7.5 and 2016 *M*_w_ 7.8 (Ms 7.5) Ecuador, and the 1943 *M*_w_ 8.1 and 2015 *M*_w_ 8.3 Illapel events, which ruptured similar source areas but did not repeat in an identical manner (*42*, *62*). For example, the rare, well-recorded Tokachi-Oki events both ruptured overlapping slip regions with primarily downdip directivity, suggesting the persistence of underlying fault structures. However, only the 1952 event was initiated by an *M*_w_ 6.1 subevent and cascaded into the adjacent Akkeshi-Oki region, revealing notable differences in the initiation and termination of hierarchical rupture growth (*42*). This type of stochastic cascading rupture, initiated by a small subevent and expanding into a large-scale event, poses challenges for early warning efforts and closely parallels the 2011 *M*_w_ 9.0 Tohoku-Oki earthquake, where a tiny *M*_w_ 4.9 initiation evolved into a massive rupture across a hierarchically structured fault area that had historically hosted only much smaller earthquakes (*7*, *63*–*65*). Together, these cases offer illustrative large-event analogs that are broadly consistent with persistent fault structure shaping rupture growth, motivating the testable hypothesis that hierarchical geometric controls may also influence great earthquakes. However, establishing whether and how these controls scale from M3.2 to M5.4 events to megathrust ruptures will require systematic validation especially in other subduction zones.

Last, we acknowledge that our inferences are conditioned by where the data are best resolved and where repeaters are most readily detected. The study region lies beneath onshore and nearshore areas and primarily samples the downdip portion of the seismogenic zone at depths of ∼40 to 70 km (Fig. 1A). This spatial window, together with the dense station coverage, likely improves event detectability and catalog completeness for moderate earthquakes and enhances the resolution of hypocenter-centroid offsets. As a result, repeaters, the spatial clustering of hypocenters and centroids, measurable rupture directivity, and signatures of hierarchical fault structure may be more readily detected here than in less instrumented or more offshore segments, without necessarily implying a higher true prevalence. In contrast, the updip megathrust closer to the trench, characterized by different thermal conditions, fluid content, and coupling state, may share broadly similar underlying fault complexity but with spatially variable expressions of rupture style and hierarchical features that are underrepresented in our dataset. Along-strike variations in fault geometry, plate age, temperature, fluid conditions, and interplate coupling could further modulate the occurrence of repeaters, the persistence of hierarchical features, and preferred rupture propagation directions. Therefore, statements extending our conclusions to all megathrusts or to great earthquakes should be viewed as plausible but currently untested extrapolations, which require validation using comparable analyses in other subduction zones and improved offshore observations that better sample the shallow interface.

## MATERIALS AND METHODS

### Selection of target potential repeating sequences

In this study, we focus on repeaters located along the plate interface. Candidate repeating sequences are selected from a recent repeater catalog (*23*), which was constructed primarily on the basis of high waveform similarity and small S-P differential time, without imposing magnitude constraints. To ensure sufficient rupture size, we require each sequence to include at least one event with a magnitude ≥4.5. To identify subduction-type repeaters, we compute the cosine similarity (CS) (*66*) between the moment tensor of each event in candidate sequence and that of the 2011 *M*_w_ 9.0 Tohoku-Oki earthquake, a representative subduction-zone event, using National Research Institute for Earth Science and Disaster Resilience (NIED) moment tensor solutions. Because moment tensor solutions are not always available for smaller events, we define subduction-type sequences as those that include at least one event with a CS value exceeding 0.75 relative to the Tohoku-Oki earthquake (*13*). Ultimately, this approach yields 150 potential M ∼ 4.5 subduction-type repeating sequences with sufficient size, providing a robust foundation for subsequent quantitative analysis.

### HypoDD relocation

Given the large location uncertainties associated with previous study that adopt hypocenter locations from the Japan Meteorological Agency (JMA) catalog, where positions are determined based on P-wave and S-wave arrival times (*23*), we first refine the event locations using HypoDD (*24*). Each subduction-type potential repeating sequence is relocated using precise differential–travel time measurements obtained from waveform cross-correlation.

In our implementation, waveform cross-correlation is performed on finite time windows centered on the P- and S-wave arrivals, using 4-s windows that include a 1-s presignal. P-wave windows are extracted from the vertical component and S-wave windows from the horizontal components. When cutting waveform segments for cross-correlation, JMA phase picks are used when available; otherwise, arrival times are determined using PhaseNet (*67*). These cross-correlation measurements provide highly accurate relative travel-time differences, allowing HypoDD to substantially improve relative locations within each sequence.

HypoDD primarily constrains relative source locations rather than absolute positions (*24*). Moreover, while discrete phase picks are most directly sensitive to rupture initiation and thus hypocentral locations, traditional waveform cross-correlation differential times, computed over finite P- and S-wave windows extending beyond the initial onset, are sensitive to the overall waveform shape and therefore preferentially constrain a rupture-weighted average location. As a result, the refined locations obtained here are more appropriately interpreted as relative centroid-like reference locations rather than strict rupture-initiation points.

Given that (i) the analyzed events are interplate earthquakes expected to rupture a common plate interface at near-identical depths, and (ii) using within-sequence or off-plate-boundary location scatter of repeaters to assess relative location quality/uncertainty is a common practice in seismological monitoring [e.g., (*68*–*72*)], we use the absolute deviation from the within-sequence mean depth as a proxy for relative location uncertainty, recognizing that it reflects internal relative scatter rather than any absolute depth bias. Because vertical location uncertainties typically exceed horizontal ones in earthquake relocation (*22*, *24*, *73*), this depth scatter–based proxy likely provides a conservative (upper-bound) estimate of relative horizontal uncertainty. The resulting depth scatter–based uncertainties of relocated target repeating sequences are primarily less than 100 m (fig. S1).

As an independent, a posteriori check, we compare these depth-scatter estimates with the formal location uncertainties reported by HypoDD and find results consistent with our expectation that depth scatter provides a conservative proxy. Specifically, the formal HypoDD horizontal uncertainties are predominantly within ∼50 m, confirming that the depth-scatter proxy is conservative and likely overestimates the horizontal component of location uncertainty. By contrast, in the vertical direction, the depth scatter–based estimates are broadly comparable to the formal HypoDD uncertainties, which are mainly <100 m, supporting the use of depth scatter as a simple and practical proxy for relative location quality in our dataset and consistent with previous reports that depth errors are typically ∼two to three times horizontal ones (*24*, *73*).

For consistency with the uncertainty treatment adopted in the subsequent high-precision hypocenter-centroid relocation, we nevertheless retain the depth-scatter proxy in downstream analyses. Uncertainties associated with the HypoDD results have a negligible impact on the analyses that follow. This is because HypoDD relocation is used primarily to reduce within-sequence relative scatter and to provide robust initial locations for the subsequent hypocenter-centroid relocation. Since that follow-on step requires an intensive 3D grid search, improved initial locations substantially reduce the computational burden and enhance processing efficiency.

### Hypocenter-centroid relocation

To precisely constrain the relative positions of earthquake hypocenters and centroids, we use the newly developed NCC method (*22*). This technique involves intensive 3D grid search to identify the relative spatial offset that maximizes the cumulative waveform correlation coefficients (CCs) across all available stations and components. The method comprises three primary steps (fig. S2):

#### Relative centroid relocation

For each event pair (*i*, *j*) within the target dataset, the method performs a 3D grid search to determine the relative spatial and temporal offsets $∆x_{\mathrm{ij}}^{\text{cent}}$ and $∆\tau_{\mathrm{ij}}^{\text{cent}}$ that maximize the NCC, calculated by summing the ordinary CC across all seismic components (*l*) and stations (*n*)

$${\mathrm{NCC}}^{\text{cent}}\left(∆x_{\mathrm{ij}}^{\text{cent}},∆\tau_{\mathrm{ij}}^{\text{cent}}\right)=\sum_{\text{ln}}\mathrm{CC}\left(u_{\mathrm{iln}}^{\text{cent}},u_{\mathrm{jln}}^{\text{cent}}\right)$$ (1)

where the ordinary CC is given by

$$\mathrm{CC}\left(u_{\mathrm{iln}}^{\text{cent}},u_{\mathrm{jln}}^{\text{cent}}\right)=\frac{u_{\mathrm{iln}}^{\text{cent}}\cdot u_{\mathrm{jln}}^{\text{cent}}}{\sqrt{{|u_{\mathrm{iln}}^{\text{cent}}|}^{2}}\cdot \sqrt{{|u_{\mathrm{jln}}^{\text{cent}}|}^{2}}}$$ (2)

and the corresponding waveforms *u* for the event pair are expressed as

$$\left\{ \begin{array}{c} u_{\mathrm{iln}}^{\text{cent}}=u_{\mathrm{iln}}\left(\tau_{i}^{0}+t\left(x_{i}^{0},x_{n}\right)-∆T^{\mathrm{pre}}\right)\\ u_{\mathrm{jln}}^{\text{cent}}=u_{\mathrm{jln}}\left(\tau_{j}^{0}+t\left(x_{i}^{0}+∆x_{\mathrm{ij}}^{\text{cent}},x_{n}\right)+∆\tau_{\mathrm{ij}}^{\text{cent}}-∆T^{\mathrm{pre}}\right) \end{array}\right.$$ (3)

Here, the superscript *cent* denotes waveforms including entire P- and S-wave segments, extracted from vertical and horizontal velocity seismograms, respectively, $\tau^{0}$ is the catalog origin time, and $t\left(x^{0},x_{n}\right)$ represents the theoretical travel time from initial catalog location $x^{0}$ to station location $x_{n}$, $∆T^{\mathrm{pre}}$ indicates the presignal time, $∆x_{\mathrm{ij}}^{\text{cent}}$ is the relative centroid location, and $∆\tau_{\mathrm{ij}}^{\text{cent}}$ is the correction for centroid time difference from catalog origin time. To mitigate the effects of rupture duration differences between events of different magnitudes, a source-time function correction is applied before cross-correlation (*22*, *74*). We note that the ordinary CC is primarily sensitive to waveform shape, thus locating the centroid as the center of mass of the earthquake rupture by using the full P- and S-waveform segments (e.g., a 4-s time window) (fig. S2).

#### Relative hypocenter relocation

Following a similar procedure, relative hypocentral offsets $∆x_{\mathrm{ij}}^{\text{hypo}}$ and $∆\tau_{\mathrm{ij}}^{\text{hypo}}$ are determined by maximizing the NCC calculated using a scaled cross-correlation (${\mathrm{CC}}_{\text{scaled}}$)

$${\mathrm{NCC}}^{\text{hypo}}\left(∆x_{\mathrm{ij}}^{\text{hypo}},∆\tau_{\mathrm{ij}}^{\text{hypo}}\right)=\sum_{n}{\mathrm{CC}}_{\text{scaled}}\left(u_{\text{in}}^{\text{hypo}},u_{\mathrm{jn}}^{\text{hypo}}\right)$$ (4)

where the ${\mathrm{CC}}_{\text{scaled}}$ is defined as

$${\mathrm{CC}}_{\text{scaled}}\left(u_{\text{in}}^{\text{hypo}},u_{\mathrm{jn}}^{\text{hypo}}\right)=\frac{u_{\text{in}}^{\text{hypo}}\cdot u_{\mathrm{jn}}^{\text{hypo}}}{\text{max}\left({|u_{\text{in}}^{\text{hypo}}|}^{2},{|u_{\mathrm{jn}}^{\text{hypo}}|}^{2}\right)}$$ (5)

and the corresponding waveforms are

$$\left\{ \begin{array}{c} u_{\text{in}}^{\text{hypo}}=u_{\text{in}}\left(\tau_{i}^{0}+t\left(x_{i}^{0},x_{n}\right)-∆T^{\mathrm{pre}}+∆t_{n}\right)\\ u_{\mathrm{jn}}^{\text{hypo}}=u_{\mathrm{jn}}\left(\tau_{j}^{0}+t\left(x_{i}^{0}+∆x_{\mathrm{ij}}^{\text{hypo}},x_{n}\right)+∆\tau_{\mathrm{ij}}^{\text{hypo}}-∆T^{\mathrm{pre}}+∆t_{n}\right) \end{array}\right.$$ (6)

Here, the superscript *hypo* indicates very short waveform segments focusing on the P-wave onset (e.g., a 0.3-s time window spanning from 0.2 s before to 0.1 s after the P-wave arrival) (fig. S2) (*22*, *27*). Notice that the summation over component *l* in Eq. 1 is omitted, as only vertical-component acceleration seismograms are used, obtained by differentiating velocity waveforms to enhance onset sharpness. $∆x_{\mathrm{ij}}^{\text{hypo}}$ is the relative hypocenter location, $∆\tau_{\mathrm{ij}}^{\text{hypo}}$ is the correction for the hypocentral time difference from catalog origin time, and $∆t_{n}$ is a station-dependent term used to capture a waveform segment that maximizes P-wave onset similarity through scaled cross-correlation ${\mathrm{CC}}_{\text{scaled}}$ (*13*). Unlike the ordinary cross-correlation approaches, the ${\mathrm{CC}}_{\text{scaled}}$ is sensitive to both waveform shape and amplitude, enabling precise detection of inter-event P-wave onset shifts (*13*, *14*) (fig. S4) and, hence, markedly improved resolution of relative hypocenter locations (*22*, *27*).

#### Joint hypocenter and centroid inversion

To directly compare rupture initiation and termination patterns, a joint inversion is performed to simultaneously solve for both hypocenter and centroid locations within a unified coordinate system. Given that smaller-magnitude events often exhibit negligible separation between hypocenter and centroid, a “hypocentroid” is introduced by merging the two locations for these events, serving as anchors in the inversion (fig. S2). Larger events retain distinct hypocenter and centroid solutions, linked to the anchors through separately determined relative location constraints. Using small events as anchors eliminates the need to directly constrain the relative locations between the hypocenters and centroids of larger events. By integrating information from both P-wave onset–focused and full waveform segments, the joint inversion refines spatial relationships among earthquake sources, yielding high-resolution insights into rupture directivity and complexity without requiring detailed slip inversions. This improvement, however, comes at the cost of substantial computational demands and the computation time scales steeply with the number of events (*27*). The effectiveness of this method has been validated through multiple approaches, including comparisons with slip inversion results (*22*, *27*). Further technical details are provided in Chang and Ide (*22*) and Chang (*27*).

Chang and Ide (*22*) pioneered the application of the NCC method to onshore and nearshore earthquake sequences in the Tohoku-Hokkaido subduction zone using a dense land–based seismic network, achieving hypocenter and centroid uncertainties below 100 m. In their study, waveform data were retrieved from land-based stations: notably the Hi-net borehole array operated by NIED, supplemented by sensors maintained by the JMA, the University of Tokyo, Tohoku University, and Hokkaido University. These instruments primarily use three-component, short-period sensors with a 1-Hz natural frequency sampled at 100 Hz.

In the present study, we expand the array to include 150 ocean-bottom seismometers of S-net. Although these instruments share the 100-Hz sampling rate, they use three-component, short-period sensors with a 15-Hz natural frequency. To recover velocity traces compatible with the Hi-net dataset, the instrument response of the S-net records has been removed. We adopt identical parameter settings and quality-control procedures to those of Chang and Ide (*22*) and therefore expect to achieve comparable or better location precision, with both hypocenter and centroid uncertainties remaining below 100 m.

This expectation is supported by histograms of absolute deviations from the mean hypocenter and centroid depths within each interplate repeater family, identified using MD and horizontal source overlap (see the next section). These deviations are predominantly less than 100 m (fig. S3), reinforcing the sub–100 m uncertainty estimate. If location uncertainty were substantially larger, the depth-deviation distributions would be expected to be markedly broader (e.g., several hundred meters or more). The consistently small deviations (fig. S3) thus indicate that the location uncertainty is indeed very limited (below 100 m), although they do not constrain absolute depth bias. The impact of location uncertainty on the robustness of our key inferences (e.g., HC distances and azimuths) is further quantified through MC simulations (see details in sections Validation of rupture directivity and Rupture repeatability analysis).

### Repeater identification

Waveform similarity has long been the primary criterion for identifying repeaters (*8*). However, an increasing body of evidence demonstrates that high similarity may result from adjacent, closely spaced sources, rather than from true repeated ruptures of the same fault patch (*31*, *75*–*77*). It is now well recognized that waveform similarity is sensitive to a variety of factors such as inter-event distance, station azimuth, velocity structure, noise level, and filtering parameters, which limits its reliability in identifying true repeaters (*30*, *31*, *78*).

To address these limitations, recent studies have increasingly adopted physics-based criteria that explicitly consider rupture-size consistency and spatial overlap, most commonly quantified using MD and overlapped source area (OSA). A systematic survey of the literature nevertheless reveals substantial variability in the threshold values adopted for both parameters. Reference (*12*) identified at least three commonly used thresholds for MD and five for OSA across previous studies, implying a wide range of possible MD-OSA threshold combinations. The specific combinations used vary among studies and are somewhat arbitrary, complicating cross-study comparisons.

Recently, (*12*) sought to place repeater identification on a more physically grounded basis by deriving theoretically motivated thresholds for MD and OSA under simple yet reasonable assumptions and validating these thresholds using real earthquake observations. They showed that true repeaters are expected to exhibit both a small MD (MD ≤ 0.3) and substantial source-area overlap, quantified by a centroid separation no greater than 0.8 times the source dimension of the larger event, corresponding to more than ∼50% overlap of rupture areas. Compared with the broad range of MD and OSA thresholds adopted in earlier studies, the proposed criterion is relatively stringent overall, although it is not the most restrictive choice possible. Several subsequent studies of natural and induced seismicity have applied or discussed this framework when distinguishing likely repeaters from neighboring events (*55*–*57*, *79*).

In this study, we adopt this physically grounded framework to identify repeaters. Specifically, two events are classified as repeaters if their MD does not exceed 0.3 and their centroid separation is no greater than 0.8 times the source dimension of the larger event (*12*). Given that the target events are interplate earthquakes, we consider only horizontal centroid separation in repeater identification. We further restrict our analysis to earthquakes with M ≥ 3.2, for which the minimum source dimensions exceed ∼100 m; as a result, the corresponding centroid-separation threshold (0.8 × source dimension) is comparable to or larger than the location uncertainties (sub–100 m), ensuring that the adopted criterion is not compromised by location errors while retaining its original physical meaning. Following prior studies, we assume an SD of 38 MPa, which is a typical value used to estimate rupture radius for repeater identification in the Tohoku-Hokkaido subduction zone (*9*, *22*, *25*–*27*). Under these criteria, we analyze 747 earthquakes with M ≥3.2 and identify 156 repeating families comprising 484 events. As an independent, a posterior check, we also evaluate waveform similarity for the resulting repeaters by computing band-pass–filtered (1 to 4 Hz) CC (*23*, *31*). The inferred similarity is consistently high: ∼85% of the pairs yield CC ≥0.95 and ∼90% yield CC ≥0.90, lending further support for the robustness of our identified repeaters.

As previously noted, these events are interplate earthquakes that are expected to originate at nearly identical depths, while vertical location uncertainty generally exceeds its horizontal counterpart. Here, we use the absolute deviations from the mean hypocenter and centroid depths within each repeater family to represent the location uncertainty. The histograms show that most of these deviations are less than 100 m (fig. S3), further supporting the reliability of the identified repeaters. Since a smaller SD is associated with a larger rupture radius, assuming a smaller SD would result in more repeaters (*31*). To assess the impact of different SD assumptions, we tested values of 10 and 3 MPa, which are commonly used in repeater identification [see the compiled table S3 in (*31*)]. With an SD of 10 MPa, we identify 165 families (527 events), and with 3 MPa, 166 families (563 events) are identified. Overall, the results are comparable (table S1).

### Nearest neighbor test

Here, our objective is not to compare the relative performance of different clustering methods or to identify an optimal one but rather to determine whether earthquake hypocenters and centroids exhibit statistically significant spatial clustering using a simple, widely applied framework without complicated assumptions. For this purpose, we adopt the NNT (*34*), a classical and robust approach that has been extensively used to characterize spatial point patterns in geophysical datasets (*27*, *80*).

The NNT essentially compares the observed mean nearest-neighbor distance, ${\bar{r}}_{\text{obs}}$, with the expected mean distance under a random (Poisson) spatial distribution. The observed mean nearest-neighbor distance is computed as

$${\bar{r}}_{\text{obs}}=\frac{\sum r}{\eta }$$ (7)

where *r* denotes the nearest distance between events and η is the total number of events analyzed. The expected mean distance under a Poisson distribution is given by

$${\bar{r}}_{\text{exp}}=\frac{1}{2\sqrt{\rho }}$$ (8)

where ρ is event density, defined as

ρ=η/s (9)

with *S* representing the study area. Following (*27*), *S* is approximated by the minimum enclosing circle encompassing all hypocenters or centroids within each sequence. The standard error of the expected distance for η events is expressed as

$$\sigma_{{\bar{r}}_{\text{exp}}}=\sqrt{\frac{4-\pi }{4\pi \rho \eta }}$$ (10)

The degree of clustering is quantified by the dimensionless ratio

$$R=\frac{{\bar{r}}_{\text{obs}}}{{\bar{r}}_{\text{exp}}}$$ (11)

where *R* <1 indicates clustering, *R* ≈1 suggests randomness, and *R* >1 implies regular spacing. The statistical significance of clustering is evaluated using the standardized *Z* score

$$Z=|\frac{{\bar{r}}_{\text{obs}}-{\bar{r}}_{\text{exp}}}{\sigma_{{\bar{r}}_{\text{exp}}}}|$$ (12)

Clustering is considered statistically significant when *Z* >1.96 and *R* <1 at the 95% confidence level. This framework provides a robust and readily interpretable means of quantifying spatial clustering in earthquake hypocenter and centroid distributions. For small sample sizes (e.g., η < 20), statistically significant departures from randomness require increasingly larger deviations of *R* from unity, reflecting reduced statistical power (*27*, *37*). Further technical details can be found in (*27*, *34*, *37*) and (*80*).

In this study, we restrict the analysis to horizontal (2D) spatial distributions, following (*27*) and (*81*). This restriction is justified because horizontal locations are generally better constrained than depths, and large depth variations are not expected for our exclusively interplate dataset (*27*). Since some sequences may be elongated along strike, the circular approximation of *S* could introduce geometry-related boundary effects and influence expected distances under the Poisson model. We therefore repeat the analysis using the minimum enclosing ellipse to define *S* as a sensitivity test; the resulting clustering statistics are overall comparable to those obtained with the circular area assumption (figs. S7 and S8).

### Validation of rupture directivity

#### Evidence from circular statistical tests

To complement the sector-based summary in Fig. 2B, we use circular statistics to test whether HC azimuths deviate from circular uniformity and to assess whether any inferred preference is robust to both finite-sample variability and plausible azimuth perturbations. HC azimuths are defined as the bearing from hypocenter to centroid (0–360°, clockwise from north). We evaluate departures from uniformity using the Rayleigh test and report the Rayleigh *p*-value together with the circular mean direction $\bar{\theta }$ and circular variance *V* (*82*, *83*), as implemented in the *astropy* library (*84*). To maintain direct comparability with Fig. 2B, we also summarize the proportions of events falling within the predefined downdip (245–335°) and updip (65–155°) sectors and estimate their sampling uncertainty via nonparametric bootstrap resampling (10,000 realizations). Using the same set of bootstrap resamples, we additionally compute bin-wise 95% confidence envelopes for the rose diagrams (fig. S10).

For the qualified event catalog (η = 639), the HC azimuth distribution is strongly nonuniform (Rayleigh *P* = 3.95 × 10^−15^), with $\bar{\theta }=295.87{}^{\circ}$ and *V* = 0.77. The small *P* value indicates a statistically significant departure from uniformity, whereas the relatively large variance *V* implies substantial azimuthal scatter rather than tight clustering around a single direction. Together, these metrics suggest a weak but coherent preference toward the downdip direction that is expressed across a broadly distributed azimuthal population. Consistent with this tendency, 44.3% of events fall within the downdip sector, whereas 21.1% fall within the updip sector (bootstrap 95% CIs: 40.4 to 48.0% and 18.0 to 24.3%, respectively) (fig. S10A). Repeaters (η = 322) show the same pattern, with significant nonuniformity (Rayleigh *P* = 3.64 × 10^−10^), $\bar{\theta }=284.52^{{}^{\circ}}$, and *V* = 0.74, and with sector fractions of 45.3% (downdip) versus 20.2% (updip) (bootstrap 95% CIs: 40.1 to 50.6% and 15.8 to 24.5%) (fig. S10B). Thus, both the qualified event catalog and the repeater subset exhibit a consistent downdip-oriented tendency under a standard circular-statistical null model despite substantial azimuthal scatter.

We next test whether this inference is sensitive to azimuth errors arising from location uncertainty. We propagate horizontal location uncertainties for both hypocenters and centroids via MC perturbations (10,000 realizations) and recompute HC azimuths for each realization. Specifically, we add independent Gaussian errors to the east and north components of each location, $e_{x},e_{y}∼𝒩\left(0,\sigma_{\mathrm{xy}}^{2}\right)$. Our baseline choice is σ_*xy*_ = 40 m for each horizontal component; under this isotropic 2D Gaussian model, the horizontal error magnitude $r=\sqrt{e_{x}^{2}+e_{y}^{2}}$ follows a Rayleigh distribution, such that $\sigma_{\mathrm{xy}}\sqrt{-2\text{ln}\left(0.05\right)}\approx 2.447\sigma_{\mathrm{xy}}\approx 98$ m, i.e., a 95% radial uncertainty of ∼100 m. In both datasets, the evidence for nonuniformity remains robust: The Rayleigh test is significant in 100% of realizations (fraction with *P* < 0.05 = 1.000), and even the upper tail of the *P* value distribution remains far below 0.05 (97.5th percentiles: 1.75 × 10^−13^ for all events; 7.60 × 10^−9^ for repeaters). MC variability in downdip and updip sector fractions is limited and remains consistent with the observed estimates (Fig. 2B), indicating that the downdip preference is not an artifact of plausible azimuth errors.

As conservative sensitivity tests, we further repeat the analysis with larger perturbation levels, σ_*xy*_ = 80 and 100 m per component (corresponding to *r*_95_ ≈ 196 and 245 m, respectively) and find that the results remain essentially unchanged. These additional runs intentionally overestimate the location uncertainty and thus serve as conservative upper-bound stress tests, verifying that our inferred predominant rupture direction is not an artifact of the assumed location-error amplitude.

#### Evidence from representative examples

In this study, we focus on the source regions of repeaters along the Tohoku-Hokkaido subduction megathrust using a 19-year dataset and find that rupture directivity is predominantly downdip. In contrast, Yoshida *et al.* (*39*), based on ∼3 years of data from approximately the same region without specifically targeting repeaters, reported that many earthquakes primarily rupture updip. While a comprehensive verification of their results is beyond the scope of our study, we can demonstrate the validity of our results by carefully examining representative examples.

Yoshida *et al.* (*39*) estimated rupture directivity based on apparent rupture durations derived from AMRFs, using nearby smaller-magnitude events as EGFs. They presented four target-EGF event pairs as representative examples in their Fig. 2. Among them, only one pair, which comprises an M4.3 target event (ID: 2019093001023142) and an M3.1 EGF (ID: 2018101521530563) and occurred in the Naka-Oki region off Ibaraki Prefecture, overlaps with our dataset (fig. S11). The M4.3 event, the largest among the four targets, was analyzed using one of the largest data volumes among the four examples in their Fig. 2 and yielded an estimated rupture direction of approximately 150°. In contrast, our NCC relocation analysis, which takes the M3.1 event as an anchor, indicates a nearly opposite rupture direction for the same M4.3 event.

To examine the discrepancy, here, we manually picked the P-wave onsets of the two events from seismograms to measure the relative location of the hypocenters. We also calculated the cross-correlation coefficient of the waveforms of two earthquakes. For P waves, we used the vertical component from 1 s before to 2 s after the P-wave arrival, and for S waves, we used the transverse component in the 18-s from the P-wave onset. The lag times corresponding to the peak of each cross-correlation function provide the differential centroid time between the two events as estimated from the P and S waves. The seismograms were band-pass filtered using a 2-to 4-s linear phase Butterworth filter before calculating cross-correlation.

Figure S11A shows the distribution of stations where P-wave onsets were clearly detected for both events, including those from the JMA, NIED Hi-net, and S-net. S-net data were rotated to vertical and transverse components based on the azimuth information provided by Takagi *et al.* (*85*), integrated from acceleration to velocity, and high-pass filtered with a 1-Hz Butterworth filter beforehand.

Figure S11B shows the estimated time differences, and fig. S11C provides example seismograms. In fig. S11B, we plot the differential P-wave onset times and the lag times of peak cross-correlations for P and S waves at stations located between 40 and 100 km from the source. These times are referenced to the origin of M3.1 event, and the differential origin time between two events. A few stations showed reversed P-wave polarities between the two events and were excluded from the comparison.

The azimuthal pattern of the differential time between manually picked P-wave onsets indicates that the M4.3 event is located east of the M3.1 event. Although the S-net data between 40° and 150° are somewhat scattered, the land stations cover azimuths over approximately 210°, supporting this conclusion. The lag times of cross-correlation, both for P and S waves, show a similar, but weaker, trend. This implies that the centroid of the M4.3 event is also located a little east of that of M3.1 event, and the centroid is the west of the initiation point. Therefore, it can be concluded that this M4.3 earthquake propagated westward, i.e., in the downdip direction, which is nearly opposite of the result of Yoshida *et al.* (*39*).

Yoshida *et al.* (*39*) present 12 additional examples in their Fig. 3. Among these, approximately half exhibit upward rupture directivity with azimuths ranging from ∼90° to 150°, while the remaining events show various orientations (e.g., northward and southward). Here, we take a closer look at three events (IDs 2019021820414510, M3.7; 2018010913432157, M4.1; and 2016090913470471, M3.8) that exhibit the strongest upward directivity (azimuths near 90°) and are constrained by the most extensive datasets. Despite the presence of dense land–based seismic networks, including borehole stations, the apparent rupture duration distributions for these events remain extremely broad and show no clear azimuthal trend, suggesting almost no resolution of rupture direction using the EGF approach. In contrast, S-net OBS data, despite generally lower signal-to-noise ratios, consistently yield shorter apparent source durations, resulting in the observed “upward” rupture propagation. This discrepancy may indicate a potential bias between land-based and seafloor observations in the EGF approach adopted by Yoshida *et al.* (*39*). By comparison, our NCC relocation is not affected by such bias, as demonstrated by the validated pair IDs 2019093001023142 (M4.3) and 2018101521530563 (M3.1) (fig. S11). We note that only one M5.1 event (ID 2018033008173366) in Fig. 3 of Yoshida *et al.* (*39*) overlaps with our dataset. In this case, Yoshida *et al.* primarily used land-based data. Although the apparent rupture duration distribution remains somewhat broad, a weak azimuthal trend suggests southward directivity, consistent with our result.

The EGF approach adopted by Yoshida *et al.* (*39*) relies heavily on selecting EGF events located in close proximity to the target event to effectively suppress site and path effects in the recorded seismograms (*58*, *86*). This method also assumes that the EGF event has a simple source process. However, it is increasingly recognized that even small earthquakes can exhibit complex rupture behavior (*87*–*91*), which may contribute to the observed discrepancies. Furthermore, accurately estimating apparent rupture durations is essential for directivity determination but remains challenging, particularly for complex AMRFs with multiple pulses (*33*, *39*, *58*, *92*, *93*).

### Rupture repeatability analysis

To address the lack of a quantitative framework for evaluating the consistency of earthquake rupture characteristics, we define three fundamental metrics corresponding to rupture direction, size, and source location (i.e., hypocenter and centroid). Building upon these, we introduce a unified RSI to enable consistent interpretation across different rupture attributes.

#### HC azimuth

To quantify the directional clustering of rupture azimuths, we use the smallest circular range (SCR), a robust metric that represents the shortest circular arc encompassing all rupture directions (*94*). A smaller SCR indicates stronger directional alignment, and vice versa. This method operates directly on azimuthal data expressed in degrees within the range [0°, 360°).

The calculation proceeds as follows. First, all rupture azimuths {θ_1_, θ_2_, θ_3_,.., θ_*n*_} are sorted in ascending order such that

$$\theta_{\left(1\right)}\leq \theta_{\left(2\right)}\leq \theta_{\left(3\right)}\leq \ldots \leq \theta_{\left(n\right)}$$ (13)

Next, the angular gaps between consecutive azimuths are computed as

$${∆}_{i}=\theta_{\left(i+1\right)}-\theta_{\left(i\right)}\;\text{for}\;i=1,2,3,\ldots ,n-1$$ (14)

and the final gap accounting for inherent periodicity is given by

$${∆}_{n}=360{}^{\circ}-\left[\theta_{\left(n\right)}-\theta_{\left(1\right)}\right]$$ (15)

Last, the SCR is computed as

$$\mathrm{SCR}=360{}^{\circ}-\text{max}\left({∆}_{1},{∆}_{2},{∆}_{3},\ldots ,{∆}_{n}\right)$$ (16)

A family is considered directionally coherent when SCR/360° is sufficiently small.

#### HC distance

To evaluate the internal consistency of actual rupture sizes, we examine variations in HC distances. For a given family, let $d_{i}$ denote the HC distance of the *i*th event and define

$$d_{\text{max}}=\text{max}\left(d_{1},d_{2},d_{3},\ldots ,d_{n}\right)$$ (17)

and

$$d_{\text{min}}=\text{min}\left(d_{1},d_{2},d_{3},\ldots ,d_{n}\right)$$ (18)

The normalized deviation is then computed as

$$d_{\text{norm}}=\left(d_{\text{max}}-d_{\text{min}}\right)/d_{\text{max}}$$ (19)

A family is considered to have consistent HC distances if $d_{\text{norm}}$ is sufficiently small.

#### Hypocenters and centroids

We assess spatial clustering by evaluating the distribution of either hypocenters or centroids relative to their average position. Since the analyzed events are interplate earthquakes, we focus only on the horizontal direction and define each event location as $X_{i}=\left(x_{i},y_{i}\right)$. The mean location is then given by

$$\bar{X}=\left(\sum_{i=1}^{n}X_{i}\right)/n$$ (20)

The Euclidean distance of each event from the mean is calculated as

$$D_{i}=\left\|X_{i}-\bar{X}\right\|$$ (21)

and the maximum distance $D_{\text{max}}$ across all events is denoted by

$$D_{\text{max}}=\text{max}\left(D_{1},D_{2},D_{3},\ldots ,D_{n}\right)$$ (22)

To normalize, we calculate the theoretical rupture radius $R_{\text{max}}$ of the largest-magnitude event in the family using the circular crack model

$$R_{\text{max}}={\left(\frac{7M_{0\text{max}}}{16∆\sigma }\right)}^{1/3}$$ (23)

where $M_{0\text{max}}$ is the seismic moment converted from the magnitude of the largest event, and ∆σ is the assumed SD (*31*, *95*).

A family is considered spatially coherent if $\frac{D_{\text{max}}}{R_{\text{max}}}$ is sufficiently small.

#### Rupture similarity index

To unify the interpretation of rupture repeatability across the three metrics, we define a dimensionless RSI, which ranges from 0 to 1, with higher values indicating greater internal similarity. The RSI is formulated as

RSI={1−SCR°360(RSIazi for HC azimuth)1−dmax−dmindmax(RSIdist for HC distance )1−DmaxRmax(Dmax≤Rmax;RSIhypocent for hypocentercentroid) (24)

A repeater family is classified as exhibiting repeatable rupture behavior if its RSI exceeds a specified threshold *T*. Because RSI is defined as one minus a normalized variability measure, specifying *T* is equivalent to specifying a maximum tolerated normalized variability of 1− *T* for each rupture attribute. Higher *T* values therefore impose stricter similarity requirements across rupture direction, rupture size, and source locations, whereas lower *T* values allow greater variability. This linear normalization preserves interpretability and directly links RSI thresholds to physically meaningful tolerances. For example, *T* = 0.8 allows up to 20% normalized variability, corresponding to (i) SCR/360°≤0.2 (i.e., SCR ≤ 72°), (ii) $\left(d_{\text{max}}-d_{\text{min}}\right)/d_{\text{max}}\leq 0.2$, and (iii) $D_{\text{max}}/R_{\text{max}}\leq 0.2$. Here, HC distance (i.e., $d_{\text{min}}$, $d_{\text{max}}$) provides an observation-based proxy for rupture dimension, capturing the separation between rupture initiation and termination inferred for each event. In contrast, $R_{\text{max}}$, derived from a circular crack model tied to seismic moment and an assumed SD (Eq. 23), provides a physically motivated reference length scale for normalizing the spatial scatter of hypocenters or centroids. Together, these normalizations anchor RSI thresholds to rupture-related length scales rather than to arbitrary spatial distances.

Because RSI is newly defined here, there is no established community standard for what RSI value should be regarded as “repeatable” rupture behavior. Nevertheless, RSI shares practically important characteristics with similarity measures widely used in repeater studies. Specifically, RSI is dimensionless and bounded in [0,1], with larger values indicating greater within-family consistency. In seismological practice, waveform similarity is commonly quantified using the normalized CC to characterize similarity among candidate repeating events. Although CC is formally defined on [−1, 1], repeater studies typically focus on strong positive similarity. As a result, commonly used thresholds effectively operate on a [0, 1]-like scale that emphasizes high positive correlation, and events are commonly considered “repeating” when CC exceeds a prescribed threshold [e.g., CC ≥ 0.80; see the compiled table S1 of (*31*)]. Motivated by this widely adopted convention, we adopt 0.80 as a conservative reference value for rupture repeatability. We do not rely on a single cutoff: We treat *T* as a tunable strictness parameter and systematically evaluate trends across a broad range of thresholds (e.g., *T* = 0.80 to 0.95) to ensure that our conclusions are not tied to any particular choice.

#### MC uncertainty propagation and “distinguishable repeatability”

To assess what level of repeatability is resolvable given location uncertainties, we propagate horizontal location errors through the RSI workflow using MC simulations. We start from the repeater catalog identified under our preferred SD assumption of 38 MPa and exclude families that contain any event with a HC distance <100 m. For each retained family, we perform 10,000 realizations by perturbing hypocenter and centroid locations in the horizontal plane with independent Gaussian noise added to the east and north components (σ_*xy*_ = 40 m per component, corresponding to an ∼100-m 95% horizontal/radial uncertainty under an isotropic 2D error model; see section Validation of rupture directivity), and recompute all RSI metrics for each realization.

We define the composite similarity as the most stringent constraint across rupture attributes

$${\mathrm{RSI}}_{\text{comp}}=\text{min}\left({\mathrm{RSI}}_{\text{azi}},{\mathrm{RSI}}_{\text{dist}},{\mathrm{RSI}}_{\text{hypo}},{\mathrm{RSI}}_{\text{cent}}\right)$$ (25)

so that a family is classified as repeatable only when all constituent aspects are jointly repeatable. To connect threshold-based statements to uncertainty, we further define an EP for the composite metric

$$\mathrm{EP}\left(T\right)=P\left({\mathrm{RSI}}_{\text{comp}}\geq T\right)$$ (26)

estimated for each family as the fraction of MC realizations with ${\mathrm{RSI}}_{\text{comp}}\geq T$. We classify families into three categories: high-confidence repeatable (EP ≥ 0.95), indistinguishable (0.05 < EP < 0.95), and high-confidence not repeatable (EP ≤ 0.05). This classification separates families that are genuinely repeatable from those that cannot be resolved as repeatable given uncertainties. Our results show that families with fully repeatable rupture behavior are very rare (fig. S16).

As sensitivity tests, we repeat the full workflow using alternative parameter choices, including repeater catalogs identified with 10 and 3 MPa, HC-distance thresholds of 0 to 500 m, and substantially larger horizontal perturbation levels (σ_*xy*_ = 80 and 100 m per component, corresponding to 95% radial uncertainties of ∼200 and ∼250 m, respectively). The resulting EP-based classifications and summary statistics remain overall comparable across these tests, indicating that our conclusions are not sensitive to these analysis choices.
