# Supplementary material for: Hierarchical repeating earthquakes with strong downward directivity
Source: Sci Adv. 2026 Sep 18;12(38):eaeb3563. doi: 10.1126/sciadv.aeb3563 (PMC13588165; doi:10.1126/sciadv.aeb3563)
Supplement: Supplementary file 1 [file sciadv.aeb3563_sm.pdf]

Science Advances
AAAS

Supplementary Materials for

Hierarchical repeating earthquakes with strong downward directivity

Dawei Gao and Satoshi Ide

Corresponding author: Dawei Gao, daweigao@qdio.ac.cn

Sci. Adv. **12**, eaeb3563 (2026)
DOI: 10.1126/sciadv.aeb3563

The PDF file includes:

Figs. S1 to S16
Table S1
Legends for data S1 and S2

Other Supplementary Material for this manuscript includes the following:

Data S1 and S2

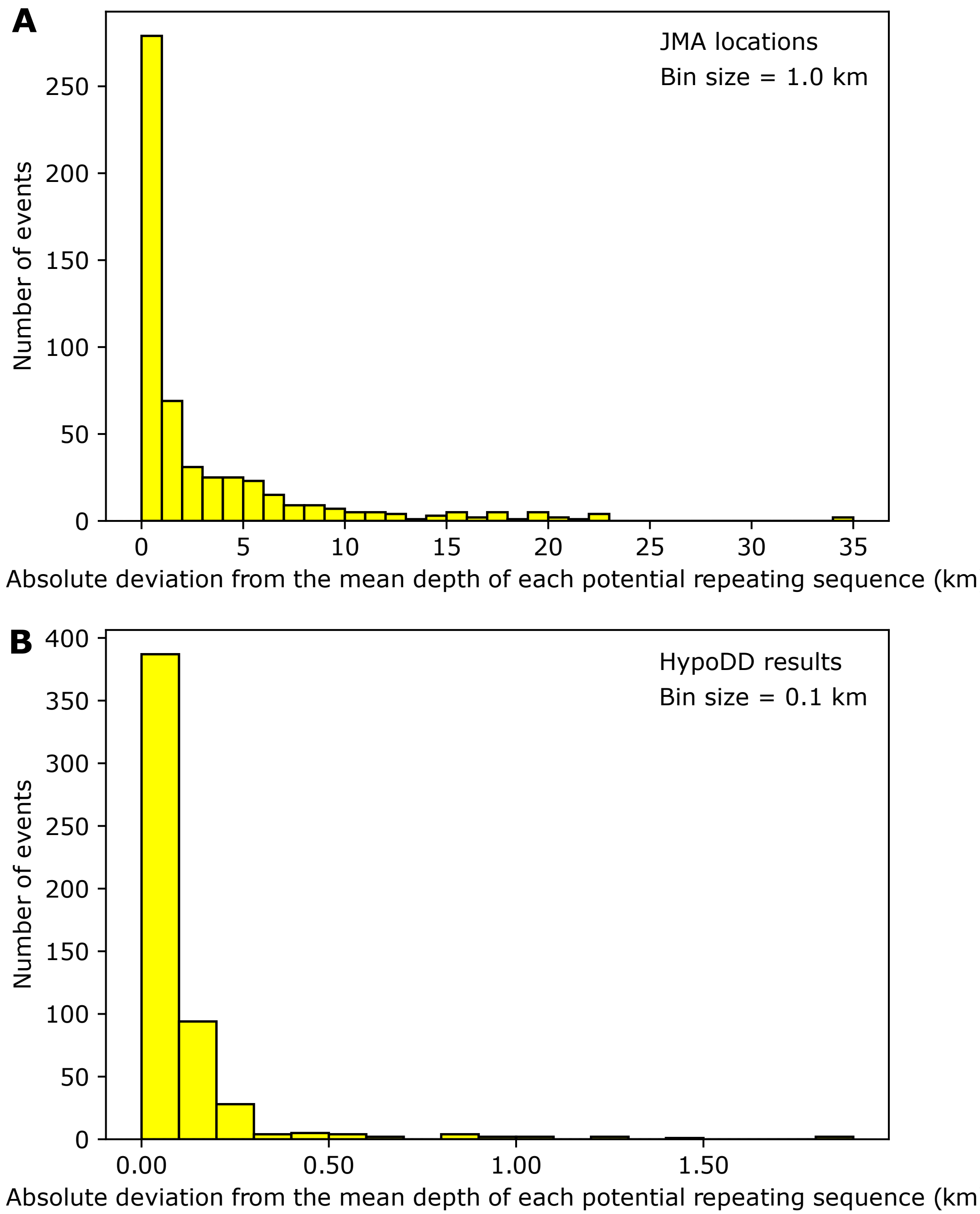

Fig. S1. Location uncertainty indicated by absolute deviation from the mean depth of each target potential repeating sequences (see Materials and Methods). (**A**), Uncertainty before HypoDD relocation. (**B**), Uncertainty after relocation.

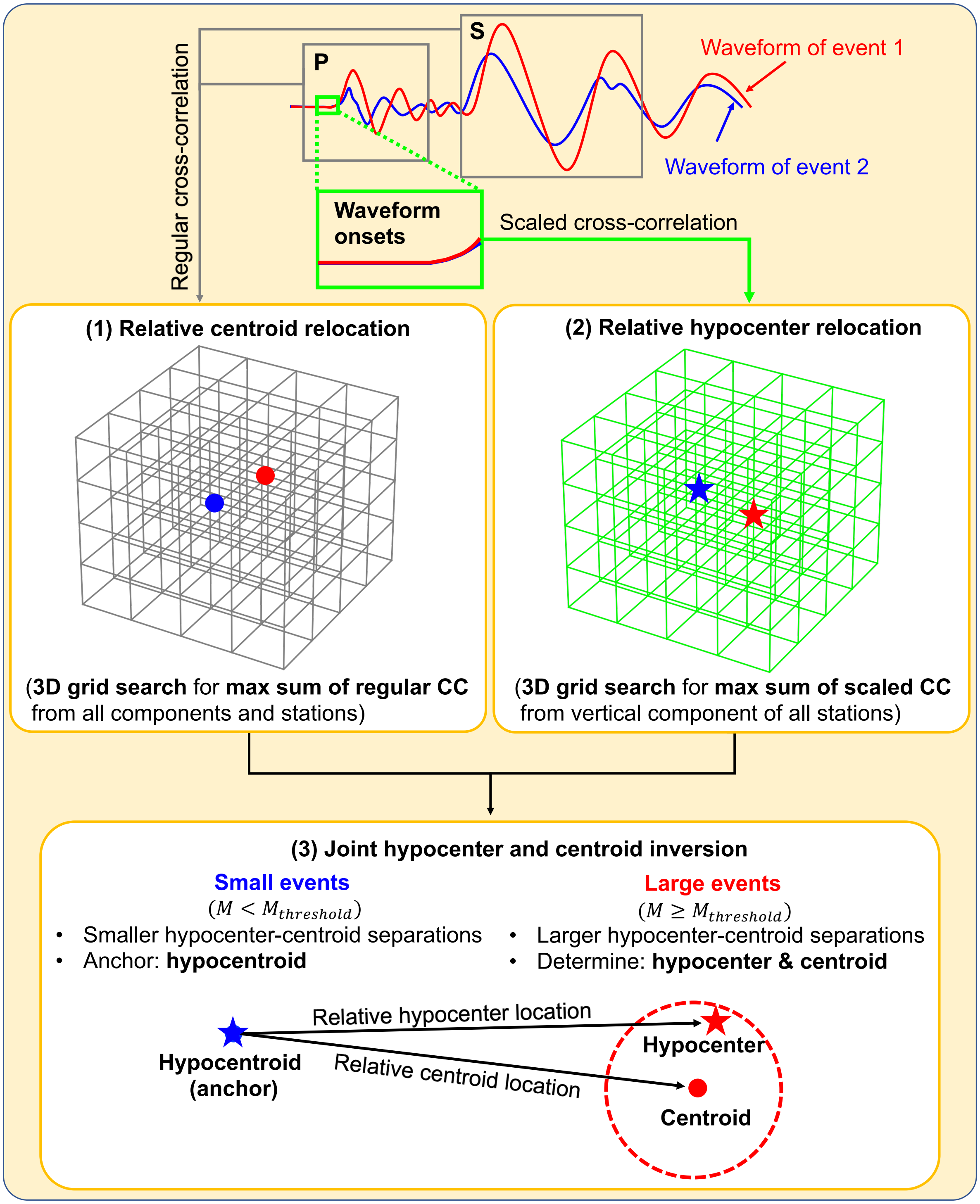

Fig. S2. Schematic illustration of the NCC method (*22*).

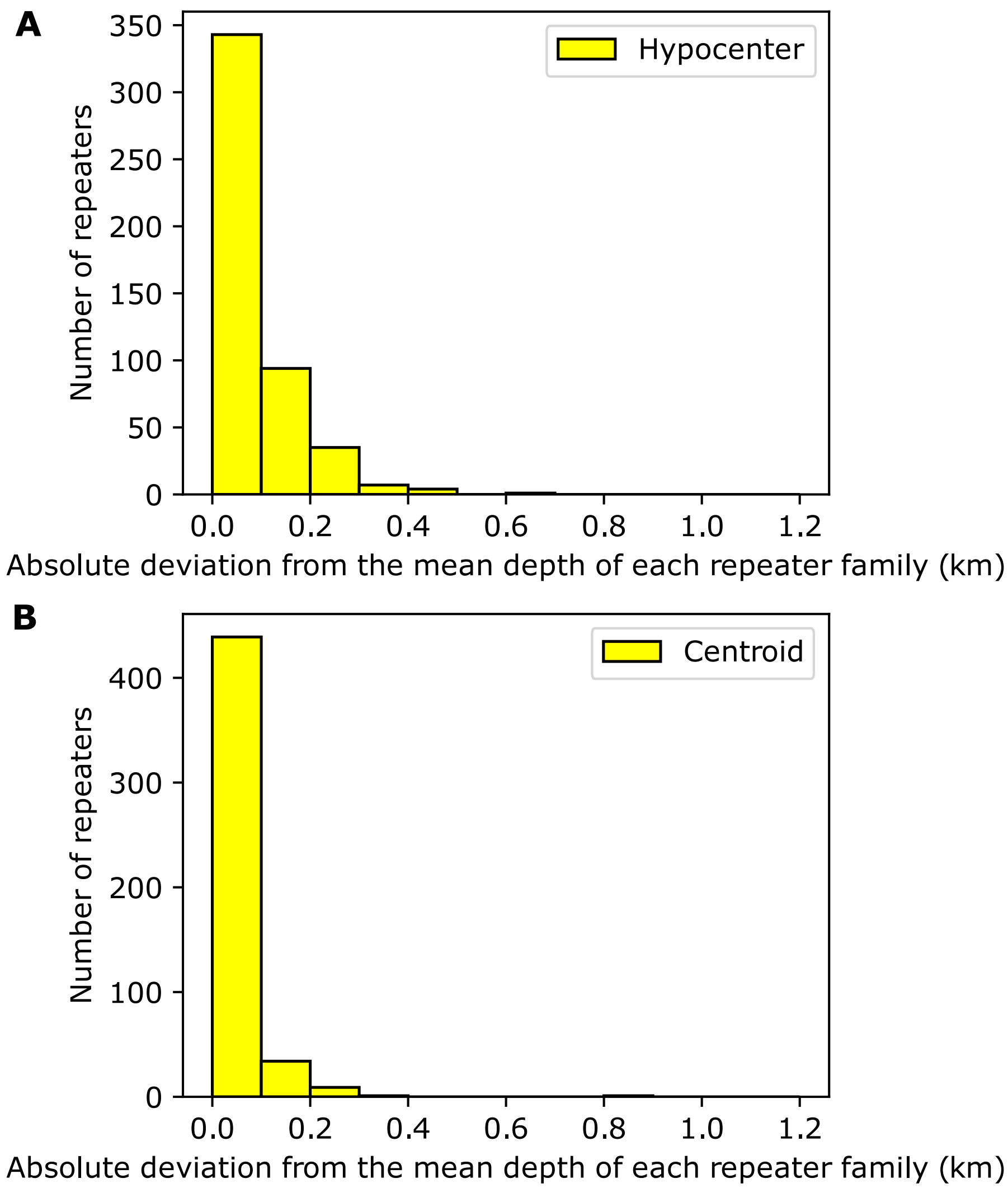

Fig. S3. Location uncertainty indicated by absolute deviation from the mean depth of each identified repeater family (see Materials and Methods). (**A**), Hypocenter. (**B**), Centroid.

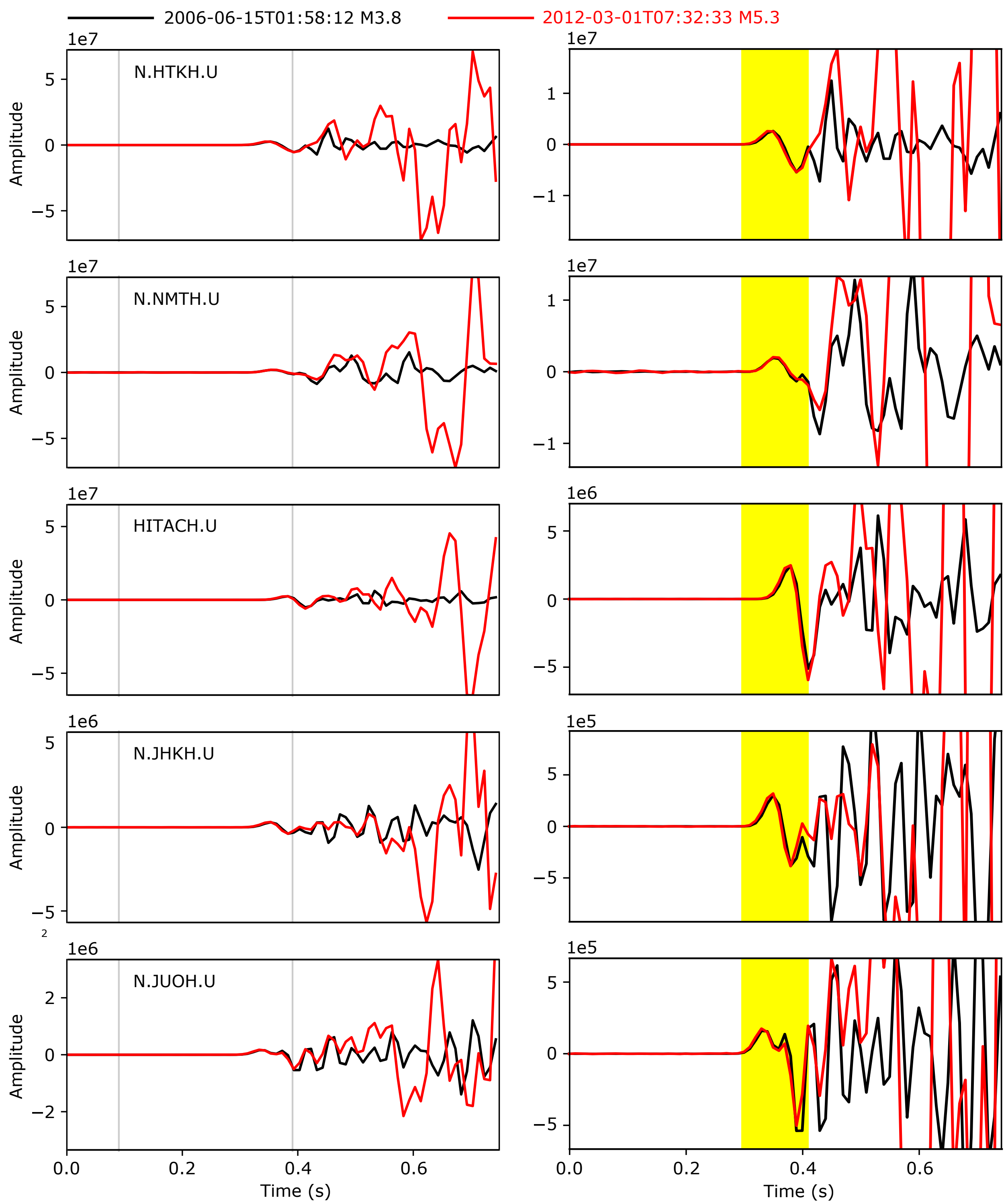

Fig. S4. Waveform examples of co-located large (red) and small (black) earthquake pairs. Left and right panels show raw waveforms at different scales. Grey vertical lines in the left panels mark the time windows used for hypocenter relocation based on scaled cross-correlation. Yellow shading in the right panels highlights the near-identical initial waveforms of co-located event pairs.

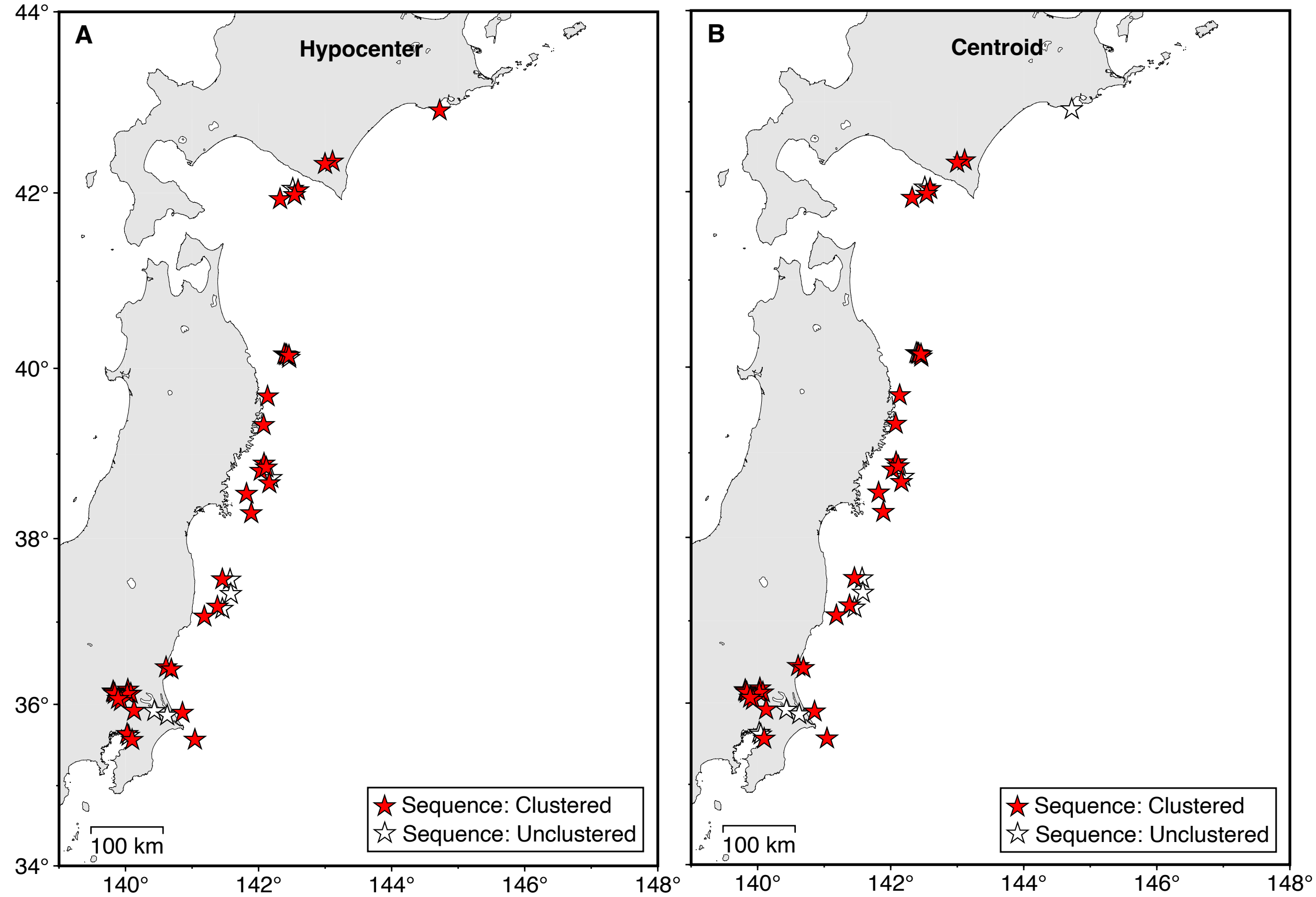

Fig. S5. Distribution of clustered sequences. (**A**), Locations of hypocenter-clustered sequences. (**B**), Locations of centroid-clustered sequences.

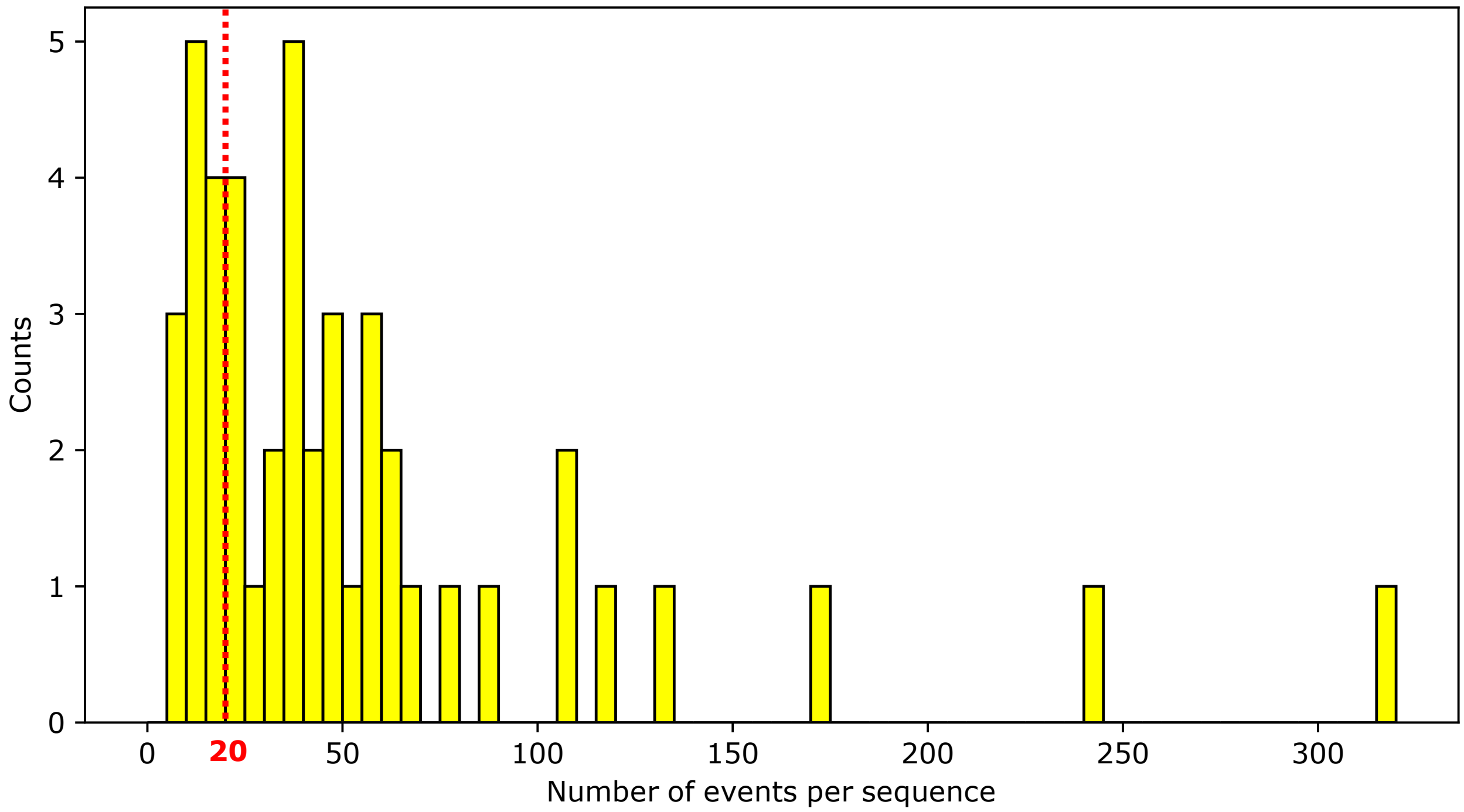

Fig. S6. Histogram showing the distribution of the number of events per analyzed earthquake sequence in the NNT analysis. The red vertical dashed line marks $\eta = 20$ for reference; sequences with fewer events ($\eta < 20$) are considered to be in the small-sample regime.

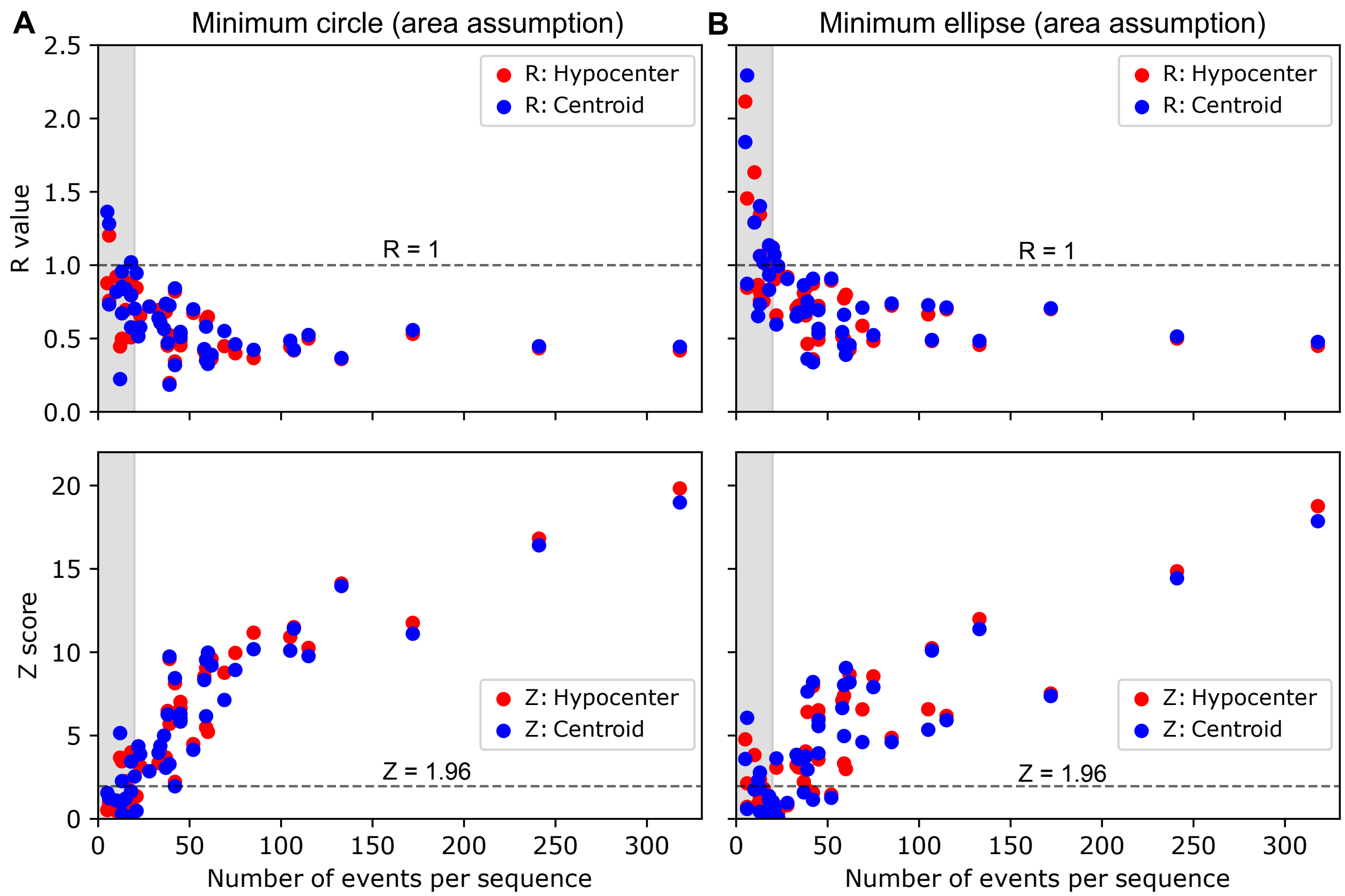

Fig. S7. Nearest-neighbour statistics versus sequence size under circle and ellipse area assumptions. Nearest-neighbour ratio R (top row) and standardized Z score (bottom row) plotted against the number of events per sequence for hypocenters (red) and centroids (blue). (**A**), minimum enclosing circle assumption; (**B**), minimum enclosing ellipse assumption. Dashed lines mark $R = 1$ (Poisson expectation) and $Z = 1.96$ (95% significance level) for reference. Grey shading denotes $\eta < 20$, highlighting the small-sample regime where interpretation should be treated with caution.

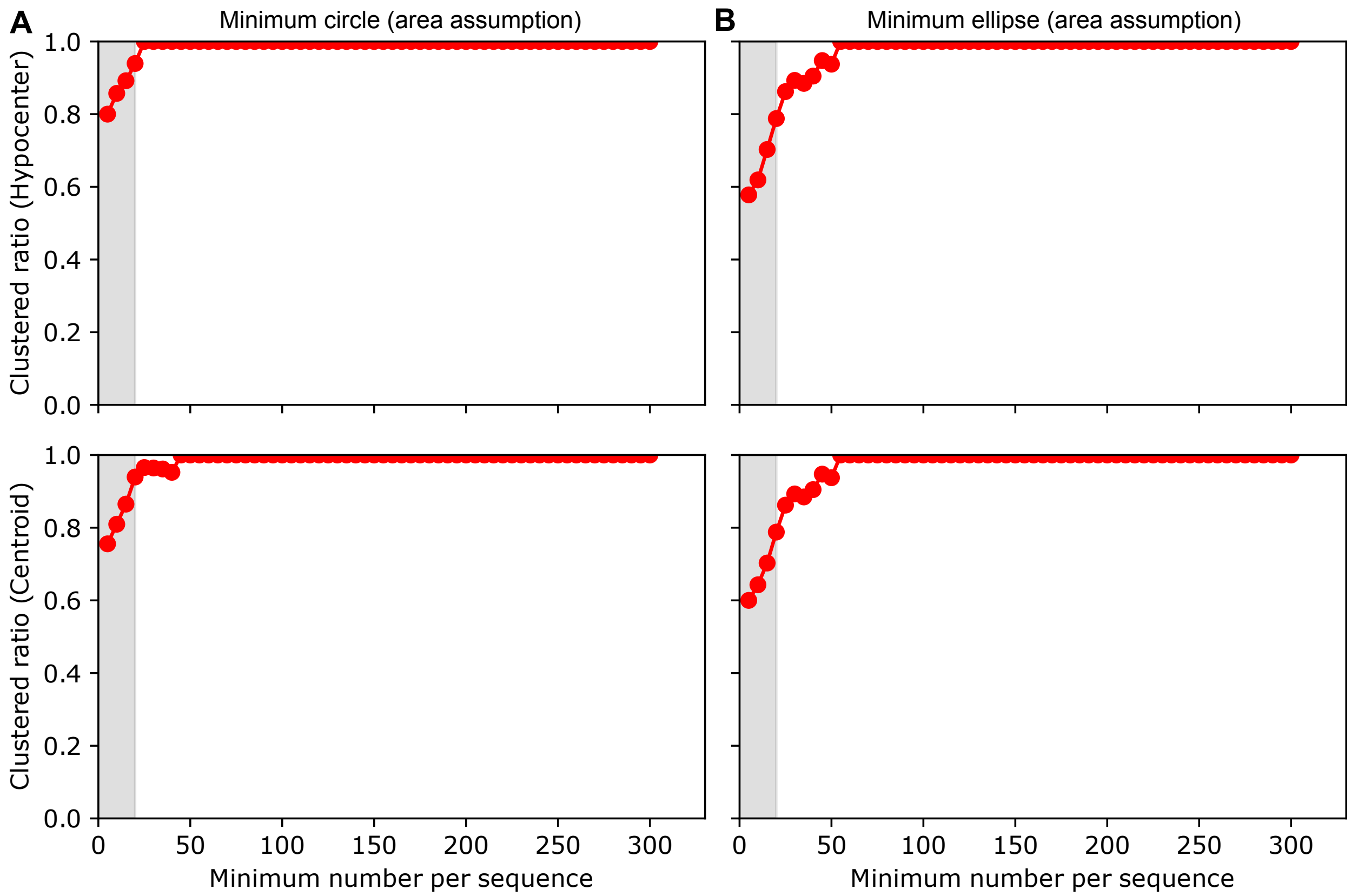

Fig. S8. Sensitivity of the clustered-sequence fraction to minimum sequence size under different study-area assumptions. Clustered ratio (fraction of sequences classified as significantly clustered by the nearest-neighbour test) versus the minimum number of events retained per sequence. Top row shows hypocenter-based results; bottom row shows centroid-based results. (**A**), study area approximated by the minimum enclosing circle. (**B**), study area approximated by the minimum enclosing ellipse. Grey shading highlights the small-sample regime (< 20 events per sequence), where interpretation should be treated with caution.

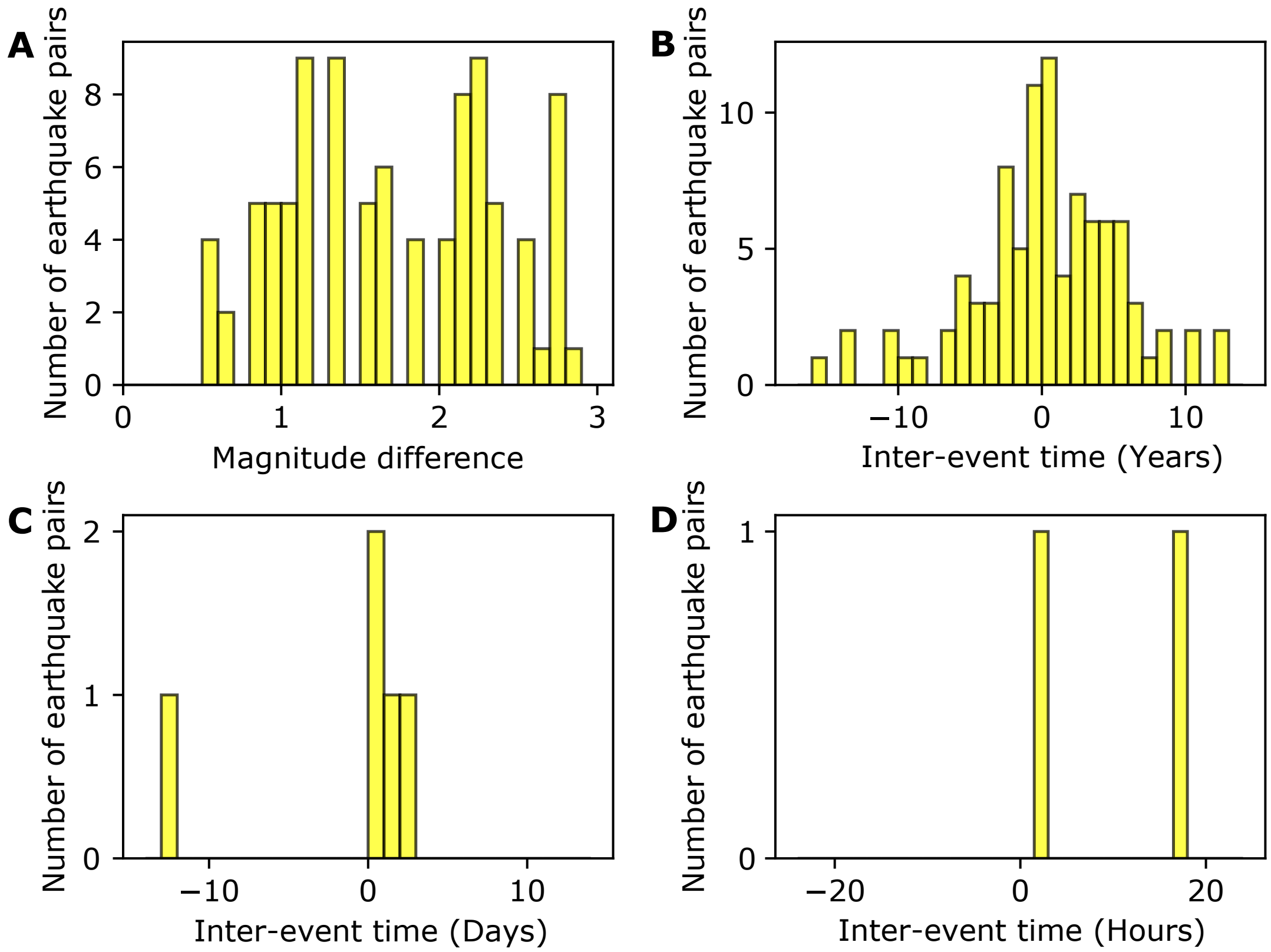

Fig. S9. Statistical properties of co-located large-small earthquake pairs when only the nearest small event occurring before and after each large earthquake is considered. (**A**), Magnitude differences. Magnitude differences are computed as absolute values and are therefore independent of event ordering within each pair. (**B-D**), Inter-event times. The inter-event time is defined as t_{small} - t_{large}: negative (positive) values indicate that the small earthquake occurred before (after) the corresponding large event.

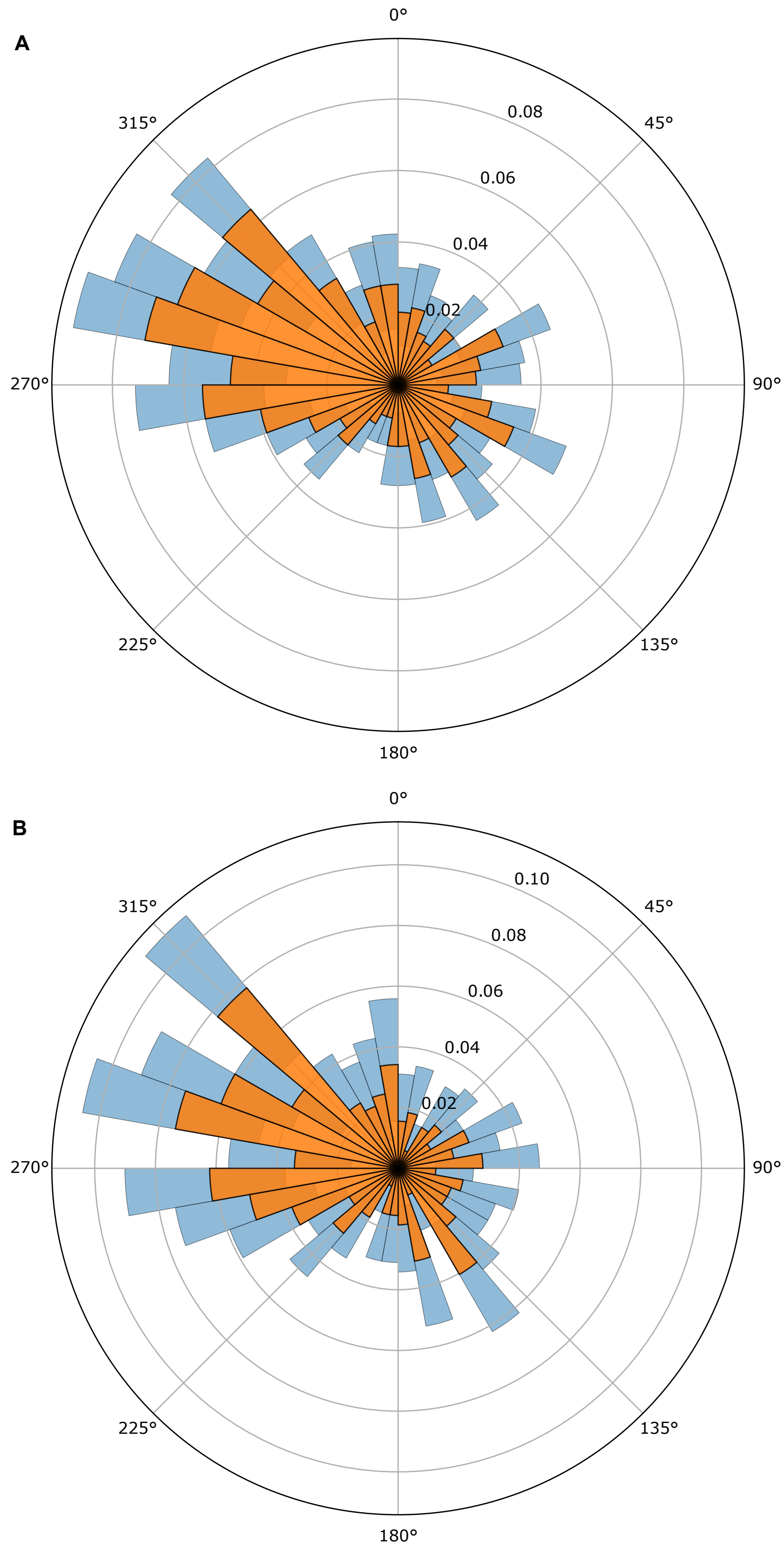

Fig. S10. Azimuthal distributions of HC directivity. (**A**), Qualified-event catalog. (**B**), Repeater subset. Polar histograms show HC directivity azimuths (0° = North; clockwise positive) binned at 10° intervals. Orange bars denote the bootstrap median fraction of events in each bin, and the light-blue shaded envelope indicates the 95% bootstrap confidence interval (2.5-97.5 percentiles), capturing finite-sample (sampling) uncertainty. Radial tick labels denote the fraction of events per bin.

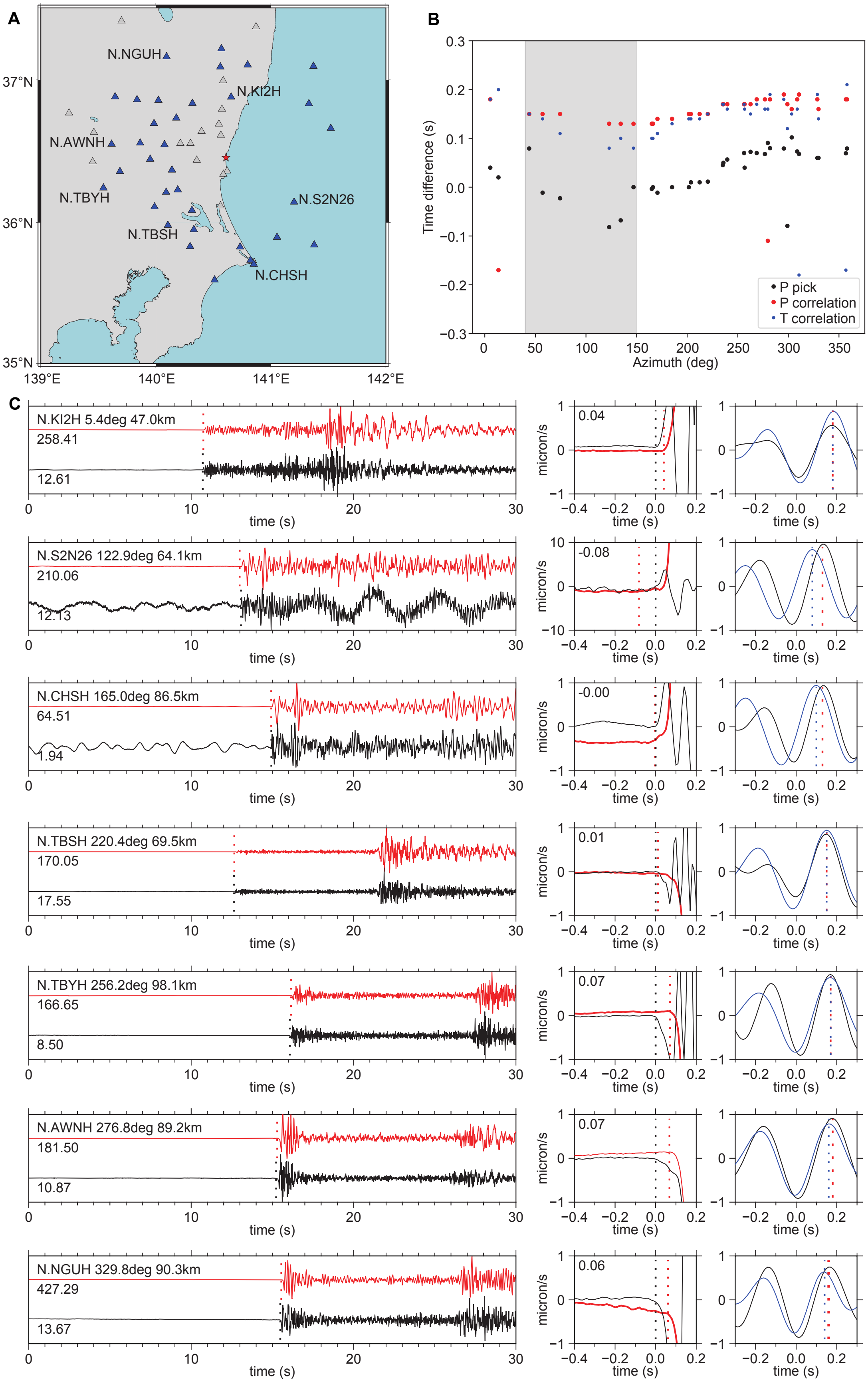
A
N.NGUH
N.KI2H
N.AWNH
N.TBYH
N.S2N26
N.TBSH
N.CHSH
37°N
36°N
35°N
139°E
140°E
141°E
142°E
B
Time difference (s)
Azimuth (deg)
P pick
P correlation
T correlation
C
N.KI2H 5.4deg 47.0km
258.41
12.61
0.04
N.S2N26 122.9deg 64.1km
210.06
12.13
-0.08
N.CHSH 165.0deg 86.5km
64.51
1.94
-0.00
N.TBSH 220.4deg 69.5km
170.05
17.55
0.01
N.TBYH 256.2deg 98.1km
166.65
8.50
0.07
N.AWNH 276.8deg 89.2km
181.50
10.87
0.07
N.NGUH 329.8deg 90.3km
427.29
13.67
0.06
time (s)
micron/s

Fig. S11. Evidence of down-dip directivity in the M4.3 event (2019093001023142), using the M3.1 event (2018101521530563) as a reference. (**A**), Map showing the distribution of stations used in this analysis. Among all available stations (triangles), those plotted in panel B are shown in blue. Station codes are also labeled for those shown in panel C. Red stars represent the M4.3 and M3.1 events. (**B**), Azimuthal distribution of the differential P-wave onset times (black), and the lag times of the maximum cross-correlation for P-waves (red) and S-waves (blue). (**C**), Examples of seismic waveforms. Left: Vertical waveforms of the M4.3 (red) and M3.1 (black) events, with the manually picked onsets indicated by vertical dotted lines. Time is measured from the catalog origin time. Center: Enlarged view of the left panel around the P-wave onset. Right: Cross-correlation functions for the P-wave (black, with the peak time marked by a red dotted line) and S-wave (blue, with the peak time marked by a blue dotted line).

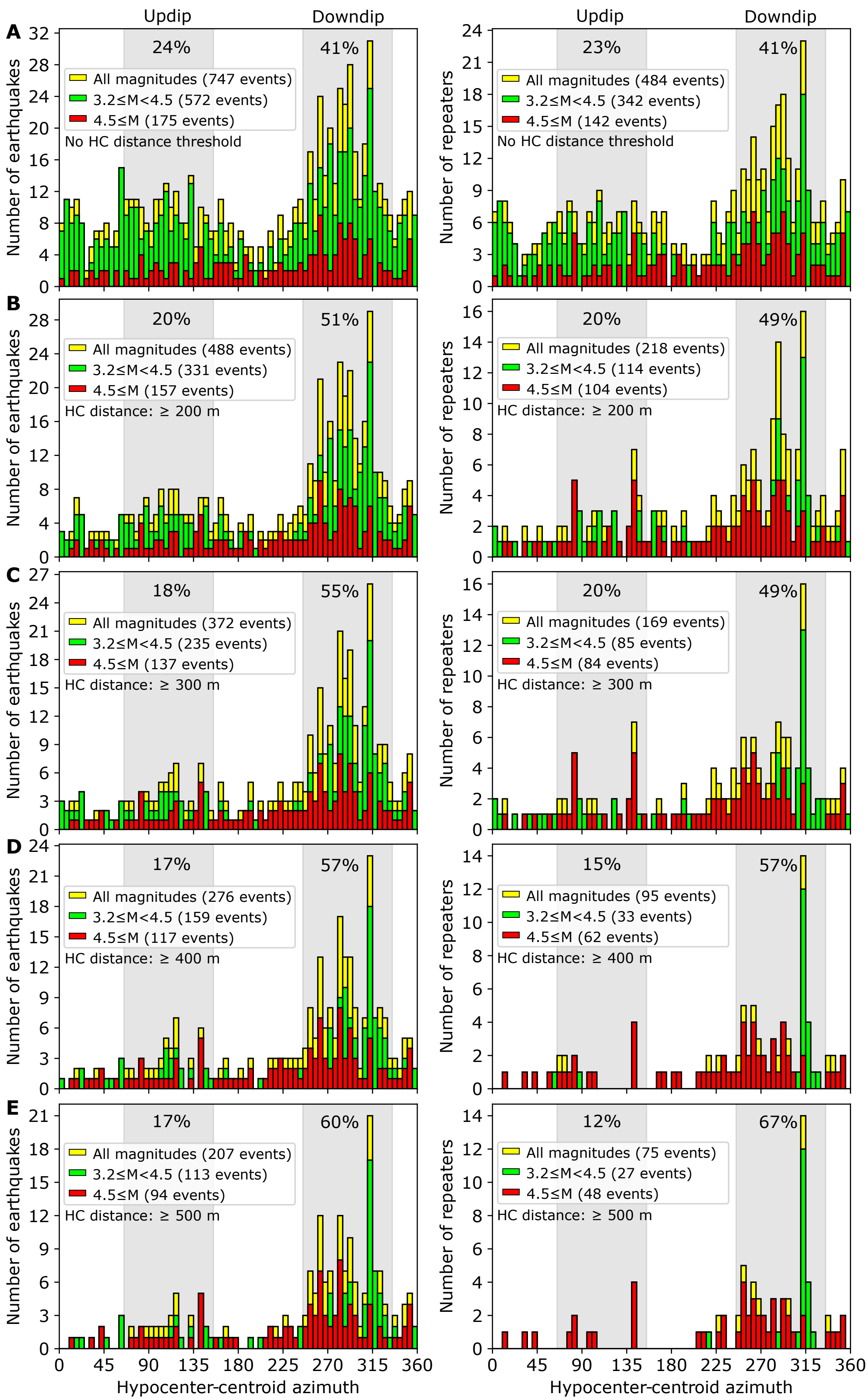

Updip
Downdip
A
24%
41%
All magnitudes (747 events)
3.2≤M<4.5 (572 events)
4.5≤M (175 events)
No HC distance threshold
Number of earthquakes
23%
41%
All magnitudes (484 events)
3.2≤M<4.5 (342 events)
4.5≤M (142 events)
No HC distance threshold
Number of repeaters
B
20%
51%
All magnitudes (488 events)
3.2≤M<4.5 (331 events)
4.5≤M (157 events)
HC distance: ≥ 200 m
20%
49%
All magnitudes (218 events)
3.2≤M<4.5 (114 events)
4.5≤M (104 events)
HC distance: ≥ 200 m
C
18%
55%
All magnitudes (372 events)
3.2≤M<4.5 (235 events)
4.5≤M (137 events)
HC distance: ≥ 300 m
20%
49%
All magnitudes (169 events)
3.2≤M<4.5 (85 events)
4.5≤M (84 events)
HC distance: ≥ 300 m
D
17%
57%
All magnitudes (276 events)
3.2≤M<4.5 (159 events)
4.5≤M (117 events)
HC distance: ≥ 400 m
15%
57%
All magnitudes (95 events)
3.2≤M<4.5 (33 events)
4.5≤M (62 events)
HC distance: ≥ 400 m
E
17%
60%
All magnitudes (207 events)
3.2≤M<4.5 (113 events)
4.5≤M (94 events)
HC distance: ≥ 500 m
12%
67%
All magnitudes (75 events)
3.2≤M<4.5 (27 events)
4.5≤M (48 events)
HC distance: ≥ 500 m
0 45 90 135 180 225 270 315 360
Hypocenter-centroid azimuth

Fig. S12. Histograms of HC azimuths for all earthquakes (left column) and repeaters (right column) under different distance thresholds. (**A**), Results including all events. (**B-E**), Results after excluding events (left column) or repeater families (right column) with HC distances less than 200, 300, 400, and 500 m, respectively. Repeaters are identified assuming a stress drop of 38 MPa (*9, 22, 25-27*). Results based on alternative stress drop assumptions (e.g., 3 and 10 MPa) (see Materials and Methods) are generally comparable and are not displayed for simplicity.

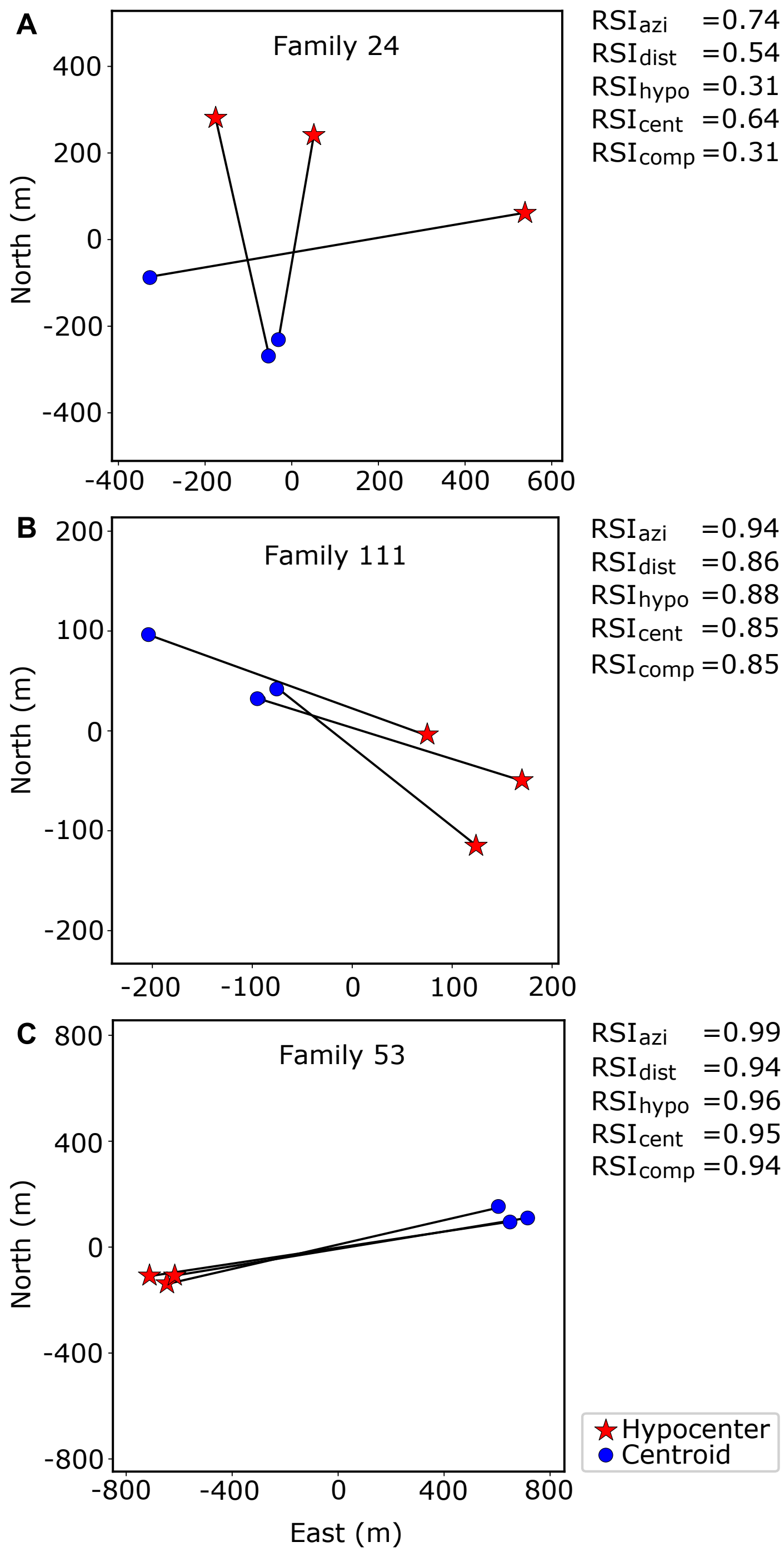

Fig. S13. Representative families spanning low to high rupture similarity. (**A-C**) Examples of repeater families with progressively higher composite similarity, from Family 24 (low), Family 111 (intermediate) to Family 53 (high). In each panel, red stars mark hypocenters and blue circles mark centroids; black segments connect each hypocenter-centroid pair, visualizing the event-specific HC vector (proxy for rupture directivity and dimension). Values at upper right report the feature-specific indices ($RSI_{azi}, RSI_{dist}, RSI_{hypo}, RSI_{cent}$) and the composite index RSI_{comp}, defined as the bottleneck similarity (minimum) across the four features.

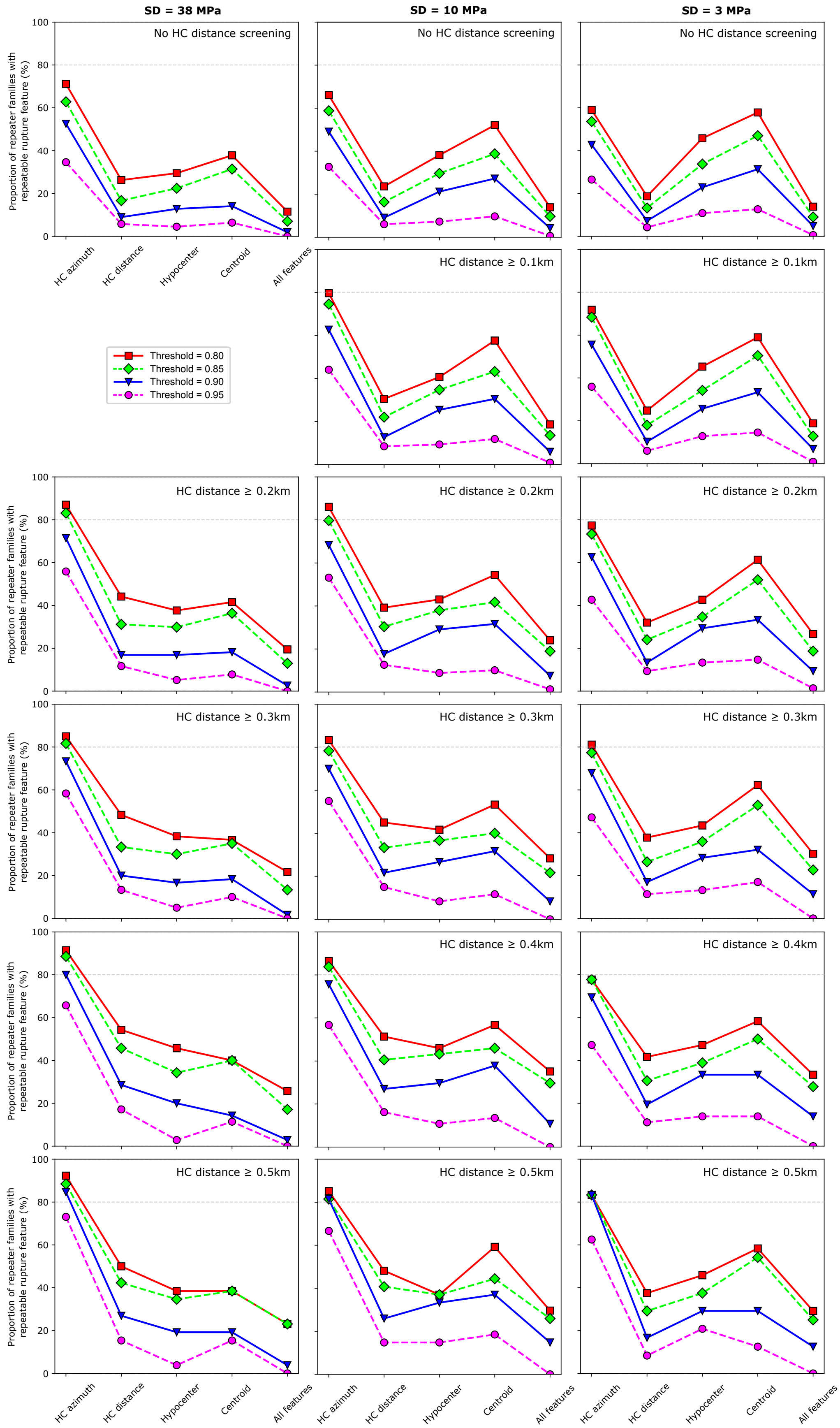
SD = 38 MPa
SD = 10 MPa
SD = 3 MPa
No HC distance screening
HC distance ≥ 0.1km
HC distance ≥ 0.2km
HC distance ≥ 0.3km
HC distance ≥ 0.4km
HC distance ≥ 0.5km
Proportion of repeater families with repeatable rupture feature (%)
Threshold = 0.80
Threshold = 0.85
Threshold = 0.90
Threshold = 0.95
HC azimuth
HC distance
Hypocenter
Centroid
All features
0
20
40
60
80
100

Fig. S14. Rupture repeatability of repeaters under different stress drop assumptions and distance thresholds. The first, second, and third columns show results assuming stress drops of 38, 10, and 3 MPa, respectively. The first row includes all repeater families. The second to sixth rows show results after excluding families that contain events with HC distances less than 100, 200, 300, 400, and 500 m, respectively.

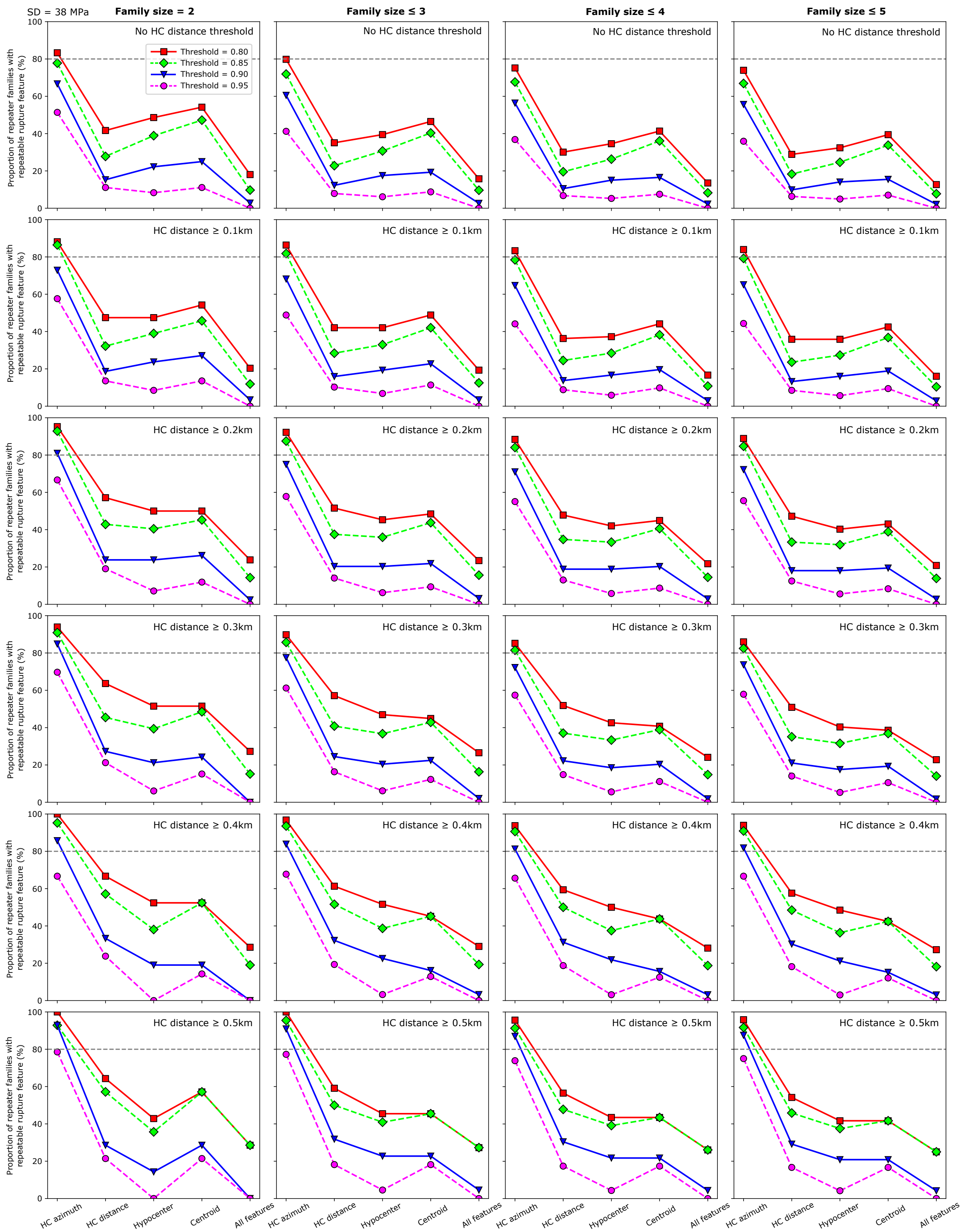

SD = 38 MPa
Family size = 2
Family size ≤ 3
Family size ≤ 4
Family size ≤ 5
No HC distance threshold
HC distance ≥ 0.1km
HC distance ≥ 0.2km
HC distance ≥ 0.3km
HC distance ≥ 0.4km
HC distance ≥ 0.5km
Threshold = 0.80
Threshold = 0.85
Threshold = 0.90
Threshold = 0.95
Proportion of repeater families with repeatable rupture feature (%)
HC azimuth
HC distance
Hypocenter
Centroid
All features

Fig. S15. Sensitivity of inferred rupture repeatability to family size and minimum HC-distance. Proportion of repeater families classified as repeatable for five aspects, evaluated for repeaters identified with a stress drop assumption of 38 MPa. Columns show results for different family-size selections (left to right: families of size = 2, and families with size ≤3, ≤ 4, and ≤ 5). Rows show the minimum HC distance required for inclusion (top to bottom: no threshold, and HC distance ≥ 0.1, 0.2, 0.3, 0.4, and 0.5 km). Results based on alternative stress drop assumptions (e.g., 3 and 10 MPa) are generally comparable and are not displayed for simplicity.

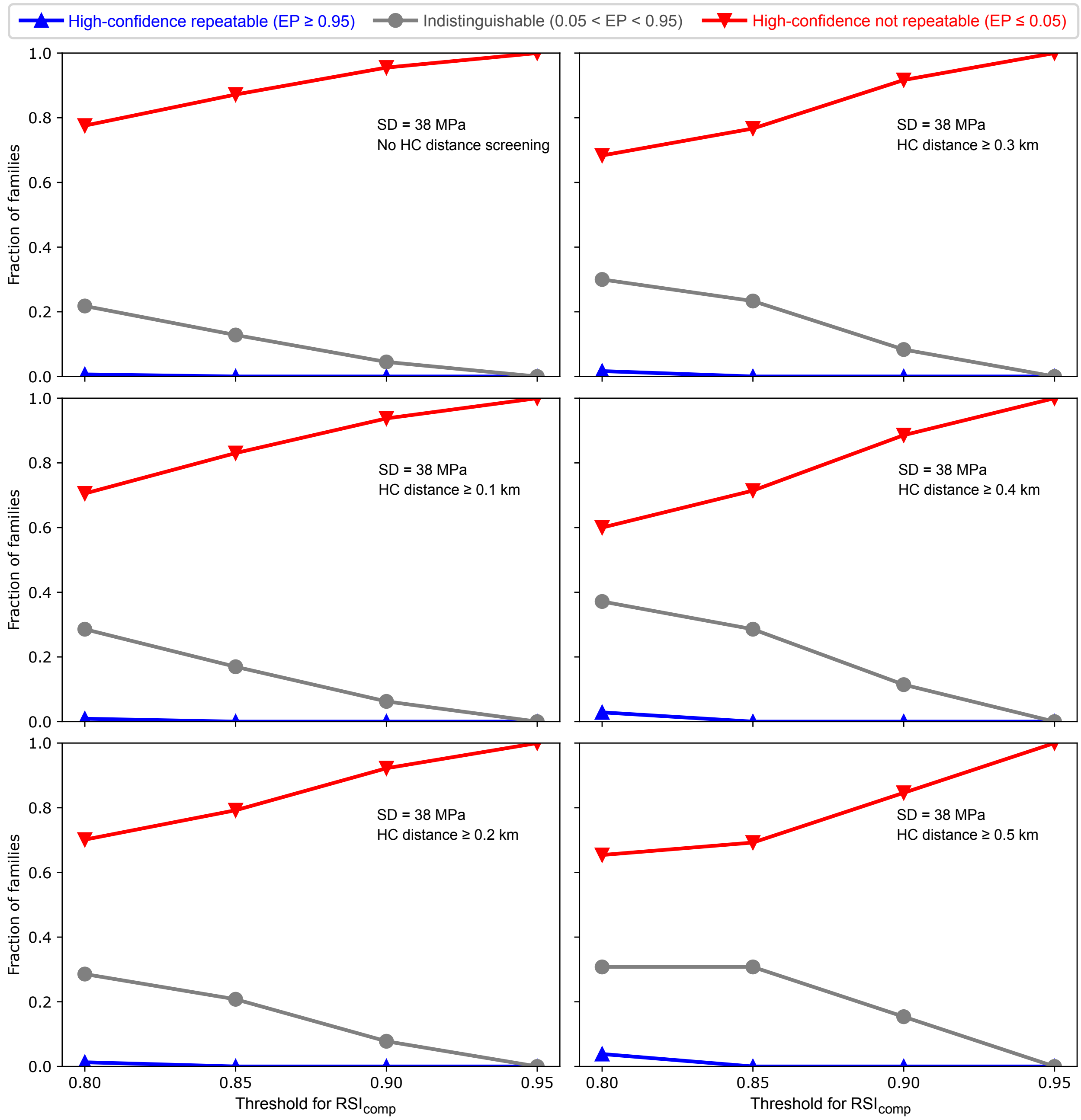

Fig. S16. Sensitivity of uncertainty-aware rupture-repeatability classification to the composite index RSI_{comp} and HC-distance screening. Fractions of repeater families classified as high-confidence repeatable (blue; EP ≥ 0.95), indistinguishable (gray; 0.05 < EP < 0.95), and high-confidence not repeatable (red; EP ≤ 0.05) are shown as a function of the composite RSI threshold applied to all four rupture features (HC azimuth, HC distance, hypocenter location, and centroid location; $T = 0.80$-0.95), based on Monte Carlo propagation of horizontal location uncertainty ($\sigma_{xy} = 40$ m). Repeater families are identified assuming a stress drop of 38 MPa, while the minimum HC-distance screening threshold varies across panels (≥ 0.0-0.5 km). Results obtained under alternative stress-drop assumptions (3 and 10 MPa) and larger perturbation levels ($\sigma_{xy} = 80$ and 100 m) are broadly comparable and are therefore omitted for clarity.

Table S1. Sensitivity of repeater statistics and rupture repeatability to assumed stress drop values

Assumed stress drop (MPa)	Number of repeater families	Number of repeater events	Proportion of repeaters	Fraction of fully repeatable families (HC distance ≥ 0.1 km)			
				$RSI_{comp} \geq 0.80$	$RSI_{comp} \geq 0.85$	$RSI_{comp} \geq 0.90$	$RSI_{comp} \geq 0.95$
3	166	563	75%	18.80%	12.82%	6.84%	0.85%
10	165	527	71%	18.64%	13.56%	5.93%	0.85%
38	156	484	65%	15.18%	9.82%	2.68%	0%

Data S1. (separate file)

Identified Repeaters

Data S2. (separate file)

Co-located Large-and-small Earthquake Pairs (i.e, Hypocenter-repeaters)
